# Morphisms of reaction networks that couple structure to function

**DOI:** 10.1186/1752-0509-8-84

**Published:** 2014-08-15

**Authors:** Luca Cardelli

**Affiliations:** 1 Microsoft Research, 21 Station Road, Cambridge CB1 2FB, UK; 2 University of Oxford Department of Computer Science, Wolfson Building, Parks Road, Oxford OX1 3QD, UK

**Keywords:** Morphisms, Chemical reaction networks, Influence networks, Biological networks

## Abstract

**Background:**

The mechanisms underlying complex biological systems are routinely represented as networks. Network kinetics is widely studied, and so is the connection between network structure and behavior. However, similarity of mechanism is better revealed by relationships between network structures.

**Results:**

We define morphisms (mappings) between reaction networks that establish structural connections between them. Some morphisms imply kinetic similarity, and yet their properties can be checked statically on the structure of the networks. In particular we can determine statically that a complex network will *emulate* a simpler network: it will reproduce its kinetics for all corresponding choices of reaction rates and initial conditions. We use this property to relate the kinetics of many common biological networks of different sizes, also relating them to a fundamental population algorithm.

**Conclusions:**

Structural similarity between reaction networks can be revealed by network morphisms, elucidating mechanistic and functional aspects of complex networks in terms of simpler networks.

## Background

### Chemical reaction networks

Chemical reaction networks provide a compact language for describing complex dynamical systems of the kind found in inorganic chemistry, biochemistry, and systems biology. They can be presented as certain graphs or as lists of reactions over a set of species. Unlike general formulations of dynamical systems in terms of differential equations, reaction networks explicitly represent *mechanism*: they present the algorithms that produce certain behaviors by a description of molecular interactions. Implicit in the simple syntax of chemical reactions are (depending on circumstances) stochastic or deterministic kinetic laws that can be used to determine the evolution of systems over time. Unravelling the exact behavior of chemical systems from the kinetic laws can be in general quite demanding; hence, attention has been dedicated to identifying functional properties of reaction networks from their structure or motifs, including questions of multistability and oscillation, and methods for transferring properties of a network to a reduced network (a vast area including [1-11]).

The aforementioned literature is focused on properties of individual reaction networks or their subnetworks. Another way to try to understand the properties of a network is to relate it to another network, perhaps a better known one, either by comparing graph structures [12-15], or more deeply by preserving kinetic features [3,10]. In this work, we identify kinetic relationships between networks that arise from network mappings, or *morphisms*. In particular, we explore the notion of *network emulation*, which allows a complex network to behave like one or more simpler networks. We show how that relationship can be determined from structural properties alone, and how it can be used to transfer system properties. As an application we obtain analytical justification of empirical relationships that have been observed in conjunction with cell cycle switch models [16].

### Influence networks

**Figure 1:**
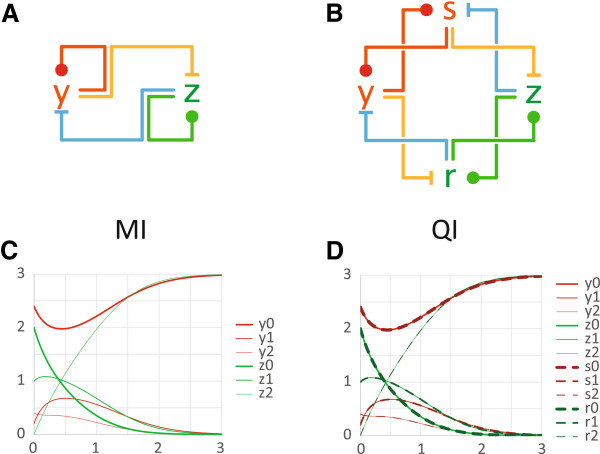
**Influence networks and network emulation. (A-B)** Each node represents an influence species. The ball-head represents an activation influence, the bar-head represents an inhibition influence, and a simple edge represents an outgoing influence to another node. Each species activates or inhibits other species catalytically. **(A)** MI network: *y* activates itself and inhibits *z*, and conversely *z* activates itself and inhibits *y*. **(B)** QI network: a more complex pattern. The colors represent mappings of species and reactions from QI to MI. **(C)** Each influence species is represented by three chemical species, yielding 6 traces for MI with arbitrarily chosen rates and initial conditions. The vertical axis is species concentrations, and the horizontal axis is time. **(D)** Via an appropriate network morphism, we can always fix rates and initial conditions for QI such that its 12 traces overlap in pairs, and exactly overlap the 6 traces of MI. Also, as a consequence, QI reaches the same steady state as MI. The rates and initial conditions for QI do not sum to, but rather copy, those of MI.

Our techniques apply to arbitrary chemical reaction networks without restrictions on reaction forms, but it will be convenient to draw examples from well-studied biological networks, which are often presented in terms of activation and inhibition correlations, or *influences. Influence networks*, such as the ones in Figure 1, are used as abstractions for more detailed biochemical interactions, capturing relationships between species that can be realized by different underlying biochemical means. The precise mechanism of these activation and inhibition influences is sometimes left informal or undetermined, but can also be characterized precisely. For example, [17,18] describe methodologies for extracting influence networks from more detailed reaction networks, and [19] systematically explores the kinetics of small influence networks.

Consider the simple influence network MI in Figure 1(A), where *y* activates itself and inhibits *z*, and where conversely *z* activates itself and inhibits *y*. It should be fairly intuitive that *y* and *z* are competing for dominance, and if *y* is ever able to fully activate itself and fully inhibit *z*, then *z* is forever inhibited, and vice versa (subject to a suitable reaction kinetics). Mutual inhibition networks arise in may areas of biology [18,20-24]; not all are this simple, and not all are reducible to the particular MI mutual inhibition pattern, but many are routinely summarized in this fashion.

The function of the network QI in Figure 1(B), however, is much harder to interpret: in MI *y* and *z* are in mutual inhibition, while in QI we have a kind of quad inhibition. We use QI as an example of a more complicated network of the kind that *could* occur in biology and whose function might not be obvious (it is in fact a simplified version of [25]). In QI, the *y, z* species seems to interact in a similar pattern as in MI; for example *z* is still (indirectly) activating itself and inhibiting *y*, and conversely. The network structure is thus suggestive of similar functionality, and one could ask whether MI and QI are in fact functionally related.

To know for sure, we need to ask if there is a similarity between the kinetics of the two networks. This question can be typically investigated by studying the kinetic equations of the networks, which can be obtained from their sets of chemical reactions. The approach we take here is instead to study the structure of the networks themselves without, at first, apparent knowledge of their kinetics. In particular we look at *morphisms* (mappings) between chemical reaction networks, including influence networks such as these. We show that these morphism can characterize functional properties and provide an explanation of kinetic similarity based on structural similarity.

## Results and discussion

### Mass action interpretation of influence networks

A *chemical reaction network* is given by a set of irreversible *reactions R* over a set of *species S*. Each reaction is written *ρ* → ^*k*^ *π*, where *ρ* are the reagents, *k* > 0 is the rate constant (we assume mass action kinetics), and *π* are the products. Both *ρ* and *π* assign a stoichiometric number to each species in *S*. For example the reaction 2*A* + *B* → ^*k*^ *B* + *C*, has reactant stoichiometric number 2 for species *A*, 1 for species *B*, and 0 for species *C*, hence *ρ*_A_ = 2, *ρ*_B_ = 1, *ρ*_C_ = 0, and similarly for *π* on the products side; *ρ* and *π* and are called *complexes*.

**Figure 2:**
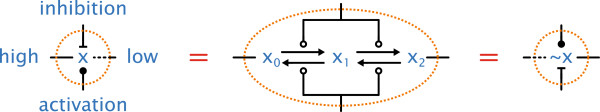
**Influence network notation.** Each influence node *x* corresponds to 3 chemical species *x*_0_, *x*_1_, *x*_2_ and four reactions, in a pattern that we call a triplet motif. The solid-edge output of a node *x* represents the activity of *x* when activated (i.e., of *x*_0_), while the dashed-edge output (which we have not needed so far) represents the activity of *x* when inhibited (i.e., of *x*_2_): in that case ‘inhibited’ is not quite the right concept, and we should think of a species *x* with two states, both having some activity. Activation and inhibition catalyze transitions between *x*_0_ and *x*_2_ through an intermediary *x*_1_ (hollow circles represent catalysis). Each node *x* can be replaced by its dual ~*x* without changing the underlying reaction network.

On the other hand, an *influence network*, such as the ones in Figure 1(A-B), is a graph of *influence nodes (species)* that range between *high* (*activated*) and *low* (*inhibited*) states, and *influence edges (reactions)* that push nodes towards activation or inhibition. Each influence node can have four terminals (Figure 2, left): high output (solid line), low output (dashed line), activation input (ball) and inhibition input (bar). Influence edges connect two such terminals in one of the four patterns: low-to-activate, low-to-inhibit, high-to-activate, and high-to-inhibit, with at most one edge of each kind for each pair of (possibly coincident) nodes.

Common characterizations of influence networks use sigmoidal (Hill or Reinitz) functions for the transitions between activated and inhibited states [19]. Here we choose an interpretation based on mass action kinetics, which results in a Hill function. Each *influence species x* (Figure 2, left) is modeled as a triplet of *chemical species*, denoted *x*_0_*, x*_1_*, x*_2_, connected in a *triplet motif* of four catalytic chemical reactions (Figure 2, center) with associated rates. The activated and inhibited states of an influence species are represented by separate chemical species: by convention *x*_0_ is the species whose concentration is high when *x* is activated and *x*_2_ is the species whose concentration is high when *x* is inhibited; *x*_1_ is an intermediary species that introduces nonlinearity in the transition, and is never otherwise connected to the rest of the network. If, for example, a species *i* is connected to the inhibition input, then the transition from *x*_0_ to *x*_1_ (arrow with hollow circle on top) represents the catalytic reaction $x_{0}+i\to^{k_{01}}\;i+x_{1}$. A duality is introduced by defining ~*x* as the species such that ~*x*_0_ = *x*_2_, ~*x*_1_ = *x*_1_, and ~*x*_2_ = *x*_0_ (Figure 2, right).

Each influence node *x* therefore corresponds to a motif of chemical reactions, and it is thus possible to translate any influence network into a chemical reaction network. Solving the mass action equations for the triplet motif at steady state yields a generalized Hill function of coefficient 2. Depending on the four reaction rates, this motif is capable of producing a range of quasi-linear, quasi-hyperbolic, and sigmoidal activation and inhibition responses (see Additional file 1).

In summary, we interpret influence networks as an unambiguous, restricted class of mass action chemical reaction networks, in a way that is not too far from common practice because it is based on an explicit mechanism that yields Hill functions. The results that we derive in Methods hold for arbitrary chemical reaction networks, and hence for all influence networks. In the main body of this paper, for exposition purposes, we concentrate on examples of influence networks, while in Additional file 2 we provide many examples of small reaction networks. The class of chemical reaction networks that we admit consists of finite sets of irreversible reactions over finite sets of species, where the reaction rates are constants and are interpreted with mass action kinetics. There are no further restrictions: in order to model open chemical systems and systems out of equilibrium, there are no assumptions about conservation of mass or energy, or about detailed balance. Note that even a chemical reaction network of this general kind can be systematically physically realized with DNA nanotechnology [26,27].

### Network emulation

Consider the mapping of species and reactions from QI to MI described in Figure 1 according to equal colors. It will not be clear at this point how this mapping was chosen, and obviously different ones are possible. It satisfies certain properties that will be clarified later, but for now we are just interested in observing some of its consequences. The *s* and *y* species of QI are mapped to the *y* species of MI, and similarly *r* and *z* are mapped to *z*. The mapping of reactions is in this case straightforward: any reaction between species of QI is mapped to a similar reaction between the corresponding species of MI according to the species mapping just described; this is called a *homomorphism*. As a result, for each reaction of MI we have two reactions of QI that map to it.

We have thus given a mapping between networks based on their structure, and we can now observe a surprising phenomenon about their kinetics. In Figure 1(C) we perform a numerical simulation of the kinetics of MI: there are 6 trajectories, 3 for *y* (the triplet *y*_0_, *y*_1_, *y*_2_) and 3 for *z*, as described in the previous section. We have chosen essentially random parameters for reaction rates and initial concentrations, and the 6 trajectories are all distinct. The MI system is inherently bistable, hence (except in degenerate cases) each trajectory converges to a maximum or a minimum. In Figure 1(D) we then simulate the QI network with a particular choice of parameters, and it appears to produce identical kinetics as MI. Actually, there are twice as many traces (and species) in QI: they exactly overlap in pairs, with each pair retracing a corresponding trajectory of MI.

The surprising fact is that, in general, QI can *emulate* MI (retrace all its trajectories) for *any* choice of parameters of MI (rates and initial concentrations). The parameters of QI that achieve this matching performance can be systematically extracted from those of MI and from the mapping of species and reactions of Figure 1(A-B). In this example, the parameter selection is straightforward, although it can be a bit more complex in general. The initial concentration of each species of QI is taken *equal* to the initial concentration of the corresponding species of MI under the species mapping, and the rate of each reaction of QI is taken *equal* to the rate of the corresponding reaction of MI under the reaction mapping. As a cautionary note, this network mapping is not a instance of coarse-graining, at least in the sense that we do not take sums of concentrations.

The fact that QI emulates MI does not preclude QI from having a richer set of behaviors outside of the initial conditions that can be derived from MI: QI does in fact have more degrees of freedom. Still, successful emulation expresses the fact that QI can be regarded as a more complicated version of MI, giving at least a partial insight in its kinetics. And if QI can emulate several unrelated networks, then multiple ‘facets’ of QI can be revealed.

The kinetic emulation property must obviously be a consequence of the kinetic equations of MI and QI, but this is not (directly) what we do here. Instead, we establish the emulation property as a consequence of the existence of a structural morphism between the networks. Network emulation has a number of consequences, which we expand on in the Conclusions. But one should already be evident: a non-trivial but purely structural mapping between networks somehow guarantees that one network can exactly, kinetically, replace another network in all possible circumstances.

### Emulation among antagonistic networks

**Figure 3:**
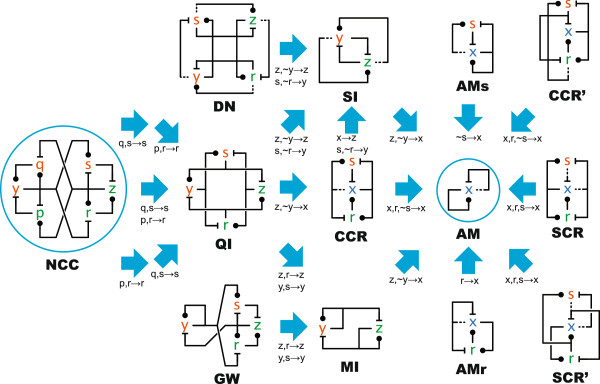
**Network morphisms.** Solid arrows are both homomorphisms and stoichiomorphisms, implying kinetic emulation similarly to Figure 1. The species mapping is indicated under each arrow; the reaction mapping is the associated homomorphic projection that simply respects the species mapping.

It turns out that it is not difficult to find examples of network emulation for influence networks. We look at a family of networks that all emulate a small mutual inhibition network, and which includes many networks commonly found in biology (Figure 3). Other families of networks can be considered as well.Each solid arrow in Figure 3 is an emulation: the source network can reproduce all the trajectories for any choice of parameters of the target network. This is not verified by checking the kinetics for equal trajectories, because we could not test all possible parameters. Instead, we construct networks morphism that satisfy the following two properties that are sufficient for emulation, as shown in Methods.

First (although this is stronger than necessary for emulation), all the morphisms in Figure 3 are *homomorphic projections*: they are obtained by collapsing certain species (as indicated under the arrows) onto species of the target network, and by letting reactions correspond according to the species mapping. In some cases we need to dualize the nodes: for example, in the morphisms leading from MI to AM we collapse ~*y* onto *x*, meaning that we map *y*_2_ onto *x*_0_, etc.; see Additional file 3 for some detailed network mappings.

Second, and most important, all the morphisms satisfy a stoichiometric relationship (*stoichiomorphism*), discussed later in this section and in Methods, that can be computed over the stoichiometric matrices and rate constants of the networks irrespectively of initial conditions.

**Figure 4:**
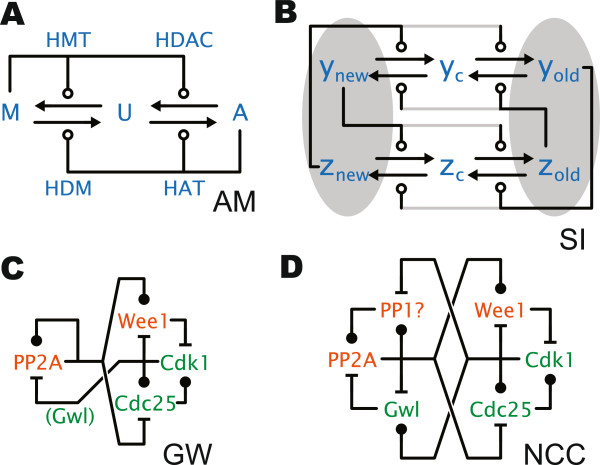
**Some biological networks corresponding to networks in Figure**3**. (A)** Epigenetic cell memory, cf. [28] Figure one. **(B)** Septation initiation network, cf. [21] Figure one. **(C)** Role of the Greatwall kinase in the G2-M cell cycle switch, cf. [29] Figure seven. **(D)** A more complete model of the G2-M switch, cf. [25] Figure three.

We now give an overview of the networks of Figure 3, and of their biological significance by reference to Figure 4. All these networks eventually morph into AM, which is a network with just three chemical species, two of which are in mutual inhibition and self activation (see Additional file 3), and converge to one of two steady states. Since all these networks can reproduce the exact trajectories of AM, by virtue of emulation we immediately obtain that all these networks are (at least) bistable.

### AM, AMr, AMs

The Approximate Majority (AM) network [16] is the quintessential population switch, with asymptotically optimal convergence speed to one of two stable steady states: convergence is achieved in *O*(log *n*) with high probability, where *n* is the number of molecules in a stochastic interpretation of the kinetics [30,31](A.4)]. Moreover, the steady states are robust to large perturbations, and they are reached quickly even in metastable conditions [30]. AMr and AMs can be generated by separately introducing indirections in the autocatalytic AM reactions, converging towards the characteristic core of a cell cycle switch network, CCR.

The exact interaction pattern of AM can be found in epigenetic switches (Figure 4(A)), where DNA histones can be in one of three states: (M)ethylated, U(nmodified), or (A)cetylated. A contiguous stretch of DNA consists of a population of histones that should be uniformly methylated or acetylated. This is achieved by the M and A states activating two proteins each that catalyze transitions between M,U,A states through the whole population. In Figure 4(A) we have expanded the AM network from Figure 3 into triplets, so that three chemical species are visible and labeled M,U,A. The resulting autocatalytic network reproduces Figure one from [28] in our notation. The known kinetics of AM implies robust uniform settling of the whole histone population into either M or A states, which is the conclusion reached in [28].

### MI

The MI network is the basic mutual inhibition network discussed in Background along with QI; its pattern can be found in many biological networks, at least in simplified form [18,20,23,24]. In genetic toggle switches, for example, the self-activation loops are usually replaced by inducers or constitutive transcription. The morphisms from QI to MI and from MI to AM are detailed in Additional file 3.

### SI

The SI network is another mutual inhibition network between two species, but with a different algorithm than MI. Two antagonists, instead of promoting themselves, are doubly active in opposing their antagonist. The SI network has exactly the same steady states as AM, while MI has an additional class of unstable steady states (see Additional file 4). QI morphs into SI, but by a less obvious mapping than into MI.

In Figure 4(B) we expand the SI network from Figure 3 into triplets. The resulting network largely matches Figure one A from [21], which is a septation initiation network: the ellipses represent the old and new spindle pole bodies that separate, and the other species are in the cytosol. Differences from [21] include the grey links, which are missing in a minimal model, or replaced by other mechanisms in more detailed models.

### CCR, GW, NCC

These networks are related to the G2-M cell cycle switch [32]. Their common structure consists of the two right-hand-side feedback loops around the species *x*,*s*,*r* (or *z*,*s*,*r*), where *x* (or *z*) is the Cdk1 protein: a cyclin-dependent kinase that has an essential role in the progress of the cell cycle. Those two loops by themselves give a close but imperfect match to AM kinetics [16], and do not technically emulate AM.

Since the basic feedback loops do not achieve optimal performance, it was suggested in [16] to consider additional known feedbacks involving the Greatwall kinase (Gwl): this gives the GW network, which was shown to perform better in simulations. We can now show analytically that GW emulates AM, and hence that it has its same switching performance. Moreover, it was independently shown [29] that the Greatwall kinase is in fact necessary for the proper biological function of the cell cycle switch. Figure 4(C) reproduces the influence network and protein assignment from [29], Figure seven: apart from an extra feedback around PPA2 that is necessary to reset the switch, that is exactly the GW network. The CCR network is a simplified version of GW where some feedbacks are short-circuited: it too emulates AM.

Figure 4(D) shows the influence network from [25], Figure three, again in our notation (a species S that is missing here just represents downstream targets of Cdk1). This is the NCC network, which has been proposed as a more complete model of the cell cycle switch, refining for example the interactions of GW. Even this rather complex network can exactly emulate AM. Moreover, the influence interactions are modeled in [25] by phosphorylation/dephosphorylation dynamics, therefore compatibly with our triplet interpretation.

NCC is a highly symmetric network, and it can emulate the equally symmetrical QI, and through it also CCR and AM. Note however that symmetry is not necessary to achieve emulation: GW is not nearly as symmetrical, and neither are AMr and AMs. It is also possible to go from NCC to QI in two steps, resulting in two less symmetrical intermediate networks before symmetry is restored (only the required collapsing of species is indicated).

NCC and GW disagree as models of the cell cycle switch (they do not emulate each other), but they agree on basic functionality. They can both emulate MI, which embodies the essence of mutual inhibition, and indirectly also AM, which embodies the essence of fast switching. Therefore, even through biological uncertainties that may be reflected in conflicting models, and even through the simplifying assumptions of modeling, we can mathematically justify what is believed to be the functional kernel of these networks. This kind of insight was used to determine that the basic cell cycle model in [16,32] may be missing some feedbacks, because it does not emulate AM and thus does not have optimal performance.

### QI

The QI network that we discussed in Background has at least two ‘facets’: it can emulate both CCR and MI, while those networks cannot emulate each other. Through CCR, QI can also emulate SI, but again MI and SI cannot emulate each other. We have no direct biological analog to offer for QI. But it can be seen as a more symmetric variation on GW where the antagonism from *z* to *y* is carried out indirectly through *s* and *r*, or else as a version of MI where self-activation is replaced by mutual activation: these are all options that are biochemically available. Both QI and GW can emulate MI, but not each other.

### DN

The DN network is a schematic Delta-Notch configuration between two neighboring cells (top and bottom halves). The tight coupling of each two nodes is due to, e.g., low-Notch (*s*) inducing high-Delta (*z*) in the same cell (top half) and high-Notch conversely inducing low-Delta because of degradation [33]. Although the basic functionality of Delta-Notch is well represented, this is an example where there is no close match with models from the literature, which all have differences in detail and miss some of the interactions. In these instances one can follow the empirical path from [16], to see whether the more realistic models still approximate the behavior of DN and AM.

### SCR, CCR’, SCR’

These are variations on other small networks, indicating that a rather large set of possibilities exists in network connectivity even after fixing the kinetics of the species. The SCR network is a version of AM where the two direct feedbacks of *x* onto itself are replaced by indirect feedbacks trough *s* and *r*. The point here is that indirections, which are common in biological networks, can (sometimes) be introduced while maintaining the emulation property. The CCR’ and SCR’ networks are just variations in the connectivity of CCR and SCR respectively, with the same number of species and the same ability to emulate AM.

### Relationships

Not all networks, even closely related ones, are connected by emulations. For example in reference to Figure 3, we can go from NCC to QI and from QI to SI in two separate steps each, but we cannot go from QI to MI in two steps. (There is no suitable morphism from CCR to MI. E.g., if we map *x* to *z* there is no target for the reactions from *x*_2_. If we merge *s* with *y* in QI and we take the homomorphic projection, we obtain a network unrelated to any in the figure.) Still, if we start from QI and we merge *s* with *y* and *r* with *z* at once, then the homomorphic projection is an emulation that leads directly to MI. This shows that there may be gradual ways to connect two networks via emulations, or there may be ‘jumps’ in complexity that, for example, would not be discovered by algorithms that attempt to identify a pair of nodes at a time. And there may be no way to relate two networks except through a common simpler network. The question of how to reach a network from another network through a sequence of emulations, is essentially the questions of how complex networks can arise from simple networks without upsetting functionality.

### Discussion: how common, useful, and brittle are network emulations?

Although we have shown many meaningful emulations morphisms, this is not the same as saying that such morphisms are common: how can we find them? The networks in Figure 3 all involve two or more antagonistic species, so that certain symmetries become apparent, and quick simulations can be used to falsify suspected symmetries. Emulations that work for some parameters can be extended to other parameters (see Methods), hence a simple strategy is to first test potential emulations with unit-rate parameters and heterogeneous initial conditions. This heuristic is imperfect, but is sufficient for all the networks in Figure 3, which are also all homomorphisms (the simplest kind of morphisms). It would seem feasible to use a tool to check all possible unit-rate homomorphisms between two networks, since we can perform emulation checks by simple matrix calculations rather than simulations (see Methods, and examples of such calculations in Additional file 3). More subtle heuristics could be devised for more subtle morphisms, and more principled algorithms for network matching could also be studied, similar to the techniques for the analysis of transition systems (see [34] and the related work referenced in [10]). It is not clear at this point how such techniques would fare on large-scale biological networks, and some notion of approximate matching would likely be required [13-15]. The examples in Additional file 2, at the level of chemical reaction networks, give some further hints about what networks may or may not be related by emulations.

Once found, the simple existence of a network emulation can reveal biologically-relevant properties of complex networks, by deriving them from known properties of simpler networks. For example, a few of the networks in Figure 3 are related to cell cycle switches, and it has been shown that the speed of cell cycle switching is important to avoid genetic instability during replication [24]. By the existence of those emulations, we immediately obtain that cell cycles switches are capable of achieving asymptotically optimal switching performance, because that is a known property of AM trajectories. This is a non-trivial consequence of emulation that speaks about the kinetics (speed of stabilization), robustness (stability of steady states), and reliability (likelihood of metastable states) of the networks, rather than just their steady state landscape. These are consequences that would be arduous to derive analytically for the larger networks, but through emulation morphisms we can take advantage of the fact that they have been derived analytically for AM [30].We have defined the notion of emulation as exact matching of trajectories between two networks, and in our examples we have chosen network patterns that produce exact kinetic emulations. This way we can be mathematically definite about the nature of the relationship, and we can derive a theory and computational methods for such morphisms. However, exact matching of trajectories is not likely to happen in practice. Even if the reaction rates were to cooperate, realistic networks always have uncertainties and minor details in network connectivity that would cause some divergence: we have discussed examples in conjunction with Figure 4 of more or less exact correspondences with biological networks. Even the notion of activation and inhibition, although widely used, is itself an approximation of underlying mechanisms that vary from case to case. And even if the true networks enjoyed a (deterministic) emulation property, stochastic mechanisms could still differentiate them.

The reality is that exact network emulation is uncommon and susceptible to perturbations: to work perfectly, it requires some adequate amount of mathematical abstraction and deviation from exhaustive biological detail. Nonetheless, the idea can be applied to imperfect situations: if the true networks do not deviate too much from the ideal networks, it is reasonable to expect that they retain their fundamental features, including the emulation relationships. This assumption should of course be tested, either with a theory of approximate network emulation that can take perturbations into account, or in absence of it, by validating approximate emulations in each particular case, for example by simulations. The latter approach was taken in [16], where it is shown that an imperfect but close emulation exists between the classical cell cycle switch [32] and AM, which is already sufficient to establish the near-optimal performance of the cell cycle switch. Moreover, a perfect emulation exists between the GW cell cycle switch [29] and AM, suggesting that a GW-like network would perform better. In general, ideal emulation relationships may suggest similar, possibly less than perfect, connections between networks. Thus, network morphisms and emulations provide a new perspective on network structure and similarity that may give helpful insights even when not perfectly realized.

### Compositionality and modularity of network emulation

The solid arrows in Figure 3 are transitive in the sense that all the relevant morphism properties formally compose (see Additional file 5). For example, QI has in general 12 distinct trajectories (4 nodes with 3 species each), and they can be made to emulate any 9 trajectories of CCR, and any 6 trajectories of SI and MI. The 6 trajectories of MI, also, can emulate any 3 trajectories of AM. It then follows by compositionality that the 12 trajectories of QI can emulate any 3 trajectories of AM.

**Figure 5:**
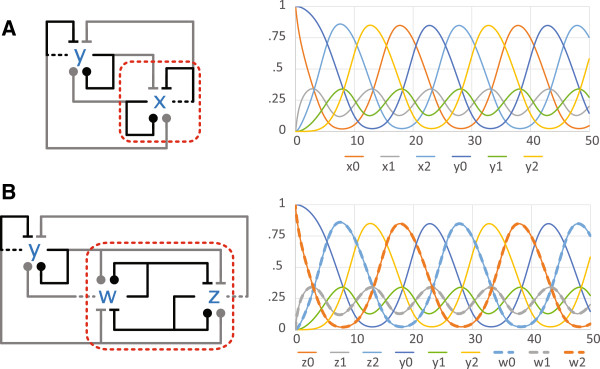
**How to replace a subnetwork of (A) and preserve emulation in (B).** The emulation from MI to AM maps *z* → *x* and ~*w* → *x*. On that basis, each influence crossing the dashed lines into *x* is replaced by a similar influence into both *z* and ~*w* (the latter is the same as an opposite influence into *w*). Each influence crossing the dashed lines out of *x* is replaced by a similar influence from the same side of either *z* or ~*w* (the latter is the same as a similar influence from the opposite side of *w*, and the same as an opposite influence from the same side of *w*).

The examples in Figure 3 are in fact more than network emulations: they are *influence network emulations* in the sense that they obey the additional constraint of mapping influence nodes to influence nodes (triplet motifs to triplet motifs). Moreover, unlike general chemical reaction networks, influence networks are (ideally) catalytic: the output side of each edge is not affected by the load at the input side. These properties conspire to guarantee a modularity property for influence networks that allows us to lift an emulation of a subnetwork to an emulation of a full network.As an example, in Figure 5(A) we have wired two AM networks to obtain a limit-cycle oscillator (the gray links between the AM’s have half the rate of the black links within the AM’s). In Figure 5(B) we have replaced the AM inside the dashed lines with an MI (since MI emulates AM), wired in a particular pattern determined by the morphism from MI to AM, ensuring that as a whole the new oscillator emulates the old oscillator. The wiring of MI ensures that the states flowing from outside to inside of the dashed lines are distributed in such a way that MI is always in the right conditions to emulate AM, and the states coming from inside to outside are thus MI states that emulate the original AM states, as far as the rest of the network is concerned.

### Summary of formal results

We now give an overview of key definitions and theorems that analytically characterize the notions of network morphism and emulation: the detailed presentation is found in Methods.

A *network morphism* from a reaction network (*S,R*) to a reaction network $\left(\hat{S},\;\hat{R}\right)$ is given by a pair of maps $m_{S}\in S\to \hat{S}$ over the species and $m_{R}\in R\to \hat{R}$ over the reactions. We are interested in two structural morphisms called reactant morphism and stoichiomorphism, and one kinetic morphism called emulation.

1. A *reactant morphism* is such that the reactants of each reaction are mapped by *m*_*R*_ according to the mapping *m*_*S*_ on species, but the reaction rates and products are unconstrained. Since network structure is related to stoichiometry, that condition turns out to be equivalent to a condition over reactant matrices:

$${m_{S}}^{T}\cdot \rho =\hat{\rho }\cdot {m_{R}}^{T}\;\text{(Definition: Reactant morphism)}$$

where the matrix ***ρ***(*s*, *r*) gives the stoichiometric number of reactant species *s* in reaction *r* of (*S, R*), and similarly for $\hat{\rho }$ of $\left(\hat{S},\;\hat{R}\right);m_{s}$ is the characteristic 0–1 matrix of *m*_*S*_, such that $m_{S}\left(s,\hat{s}\right)=\left(\mathrm{if}\;m_{S}\left(s\right)=\hat{s}\;\mathrm{then}\;1\;\mathrm{else}\;0\right)$ and similarly for ***m***_***R***_; ‘∙’ is matrix multiplication, and **-** ^**T**^ is matrix transposition.

2. A *stoichiomorphism* relates the stoichiometry of the networks in a deeper way. The *instantaneous stoichiometry* of a species *s* in a reaction *ρ* → ^*k*^*π* is defined as *φ*(*s*, *ρ* → ^*k*^*π*) = *k* · (*π*_*s*_ - *ρ*_*s*_), that is the net stoichiometry in the reaction multiplied by the reaction rate. A stoichiomorphism is then defined to satisfy:

$$\varphi \cdot m_{R}=m_{S}\cdot \hat{\varphi }\;\text{(Definition: Stoichiomorphism)}$$

where ***φ*** is the instantaneous stoichiometric matrix of (*S, R*), such that ***φ***(*s*, *r*) = *φ*(*s*, *r*); similarly for $\hat{\varphi }$.

3. An *emulation* is a morphism that relates the *differential systems* of two networks. The differential system $F\in {\mathbb{R}}_{+}^{S}\to {\mathbb{R}}^{S}$ of (*S, R*) gives for each *state*$v\in {\mathbb{R}}_{+}^{S}$ (where ***ν*** assigns concentrations to species), and for each species *s* ∈ *S*, the derivative *F*(***v***)(*s*) ∈ *ℝ* of the concentration of *s* in ***v***. *F* is defined according to the law of mass action, and similarly for $\hat{F}$ over $\left(\hat{S},\;\hat{R}\right)$. A morphism is an *emulation* if:

$$\forall \hat{v}\in {\mathbb{R}}_{+}^{\hat{S}}\;F\left(\hat{v}\circ m_{S}\right)=\hat{F}\left(\hat{v}\right)\circ m_{S}\;\text{(Definition: Emulation)}$$

This says that the derivatives of the two systems are related by *m*_*S*_. In particular, the derivatives coincide when the initial states coincide under *m*_*S*_, guaranteeing by determinism that whole trajectories coincide. This definition characterizes the coincidence of trajectories observed earlier in simulations.

Given any network morphism, the reactant morphism and stoichiomorphism conditions can be checked purely on the connectivity, stoichiometry, and rate constants of the networks. Our main theorem states that those structural conditions guarantee that the network morphism is a kinetic emulation:

*Theorem (Emulation):* If a morphisms (*m*_*S*_, *m*_*R*_) is a reactant morphism and a stoichiomorphism, then it is an emulation.

That is, for any choice of initial conditions $\hat{v}$ of $\left(\hat{S},\;\hat{R}\right)$ we can pick initial conditions $\hat{v}\circ m_{S}$ for (*S*, *R*) such that the trajectories of the two systems coincide. The rates of the two networks are coupled by the stoichiomorphism condition, but a second theorem then guarantees that we can achieve emulation also after changing rates. We show that if there is a stoichiomorphism from (*S*, *R*) to $\left(\hat{S},\;\hat{R}\right)$ and we change the rates of $\left(\hat{S},\;\hat{R}\right)$, then we can correspondingly change the rates of (*S*, *R*) so that we have again a stoichiomorphism, and hence the previous theorem applies.

### Discussion: network ‘structure’ and rate independence

The ‘structure’ of a reaction network can be understood as its connectivity, without considerations of reaction rates: in this view the network structure is a graph with no kinetic information. Interesting notions of morphisms can be developed based on this level of information [10,12,13] as well useful tools [14,15]. It is even possible to draw interesting conclusions about network kinetics just from the graph structure, but usually under some minimal assumptions about the underlying kinetics [1]. Reaction rates are usually present in the development of mathematical results, and are necessary to be able to state a rate-independence property.

More broadly, the structure of a network can be understood as the whole presentation of the network: the *state-independent* information that does not change over time and that is, in particular, independent of the initial state. Rate constants, when provided, are structural in that sense: they are part of the ‘syntax’ or raw presentation of a network, just like stoichiometric constants, before any behavioral questions are considered. They are also technically part of the mathematical structure of chemical reaction networks. It may later be possible to show that rate information does not matter for certain network properties, but note that, similarly, some connectivity information can be shown not to matter (e.g., a reaction *s* → *s*).

**Figure 6:**
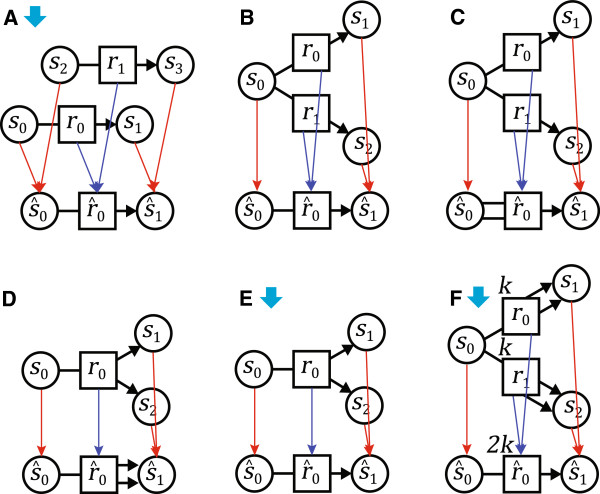
**Examples of chemical reaction network morphisms.** Circles are species and squares are reactions. Red arrows are species mappings ***m***_***S***_ and blue arrows are reaction mappings *m*_*R*_. Solid arrows indicate morphisms that are emulations. More examples are given in Additional file 2. **(A)** A simple stoichiomorphism: the species in the source reactions are distinct. In general, multiple separate copies of a system will map to it via a trivial map that is a homomorphism and stoichiomorphism. **(B)** This is a homomorphism, but is not a stoichiomorphism. For $s_{0},{\hat{r}}_{0}$: $\Sigma_{r\in {m_{R}}^{-1}\left({\hat{r}}_{0}\right)}\;\varphi \left(s_{0},r\right)=\;-2\;\neq \;-1=\;\varphi \left(m_{S}\left(s_{0}\right),{\hat{r}}_{0}\right)$. **(C)** This is a stoichiomorphism, but is not a homomorphism or a reactant morphism. *r*_0_ = *ρ* → *π* with $\rho_{s_{0}}=1$ but $m_{R}\left(r_{0}\right)=\;{\hat{r}}_{0}=\hat{\rho }\to \hat{\pi }$ with ${\hat{\rho }}_{m_{S}\left(s_{0}\right)}={\hat{\rho }}_{{\hat{s}}_{0}}=2$, so $\hat{\rho }\neq m_{S}\left(\rho \right)$ and $m_{ℛ}\left(r_{0}\right)\neq m_{S}\left(\rho \right)\to \hat{\pi }$. **(D)** This is a homomorphism but not a stoichiomorphism. For $s_{1},{\hat{r}}_{0}$: $\Sigma_{r\in {m_{R}}^{-1}\left({\hat{r}}_{0}\right)}\;\varphi \left(s_{1},r\right)=\;1\;\neq \;2=\;\varphi \left(m_{S}\left(s_{0}\right),{\hat{r}}_{0}\right)$. **(E)** This stoichiomorphism is not a homomorphisms, but is a reactant morphism. *r*_0_ = *ρ* → *π* and $m_{R}\left(r_{0}\right)=\;{\hat{r}}_{0}=\hat{\rho }\to \hat{\pi }$ with $\hat{\rho }=m_{S}\left(\rho \right)$ and $m_{R}\left(r_{0}\right)=m_{S}\left(\rho \right)\to \hat{\pi }$. **(F)** This reactant morphism is not a homomorphism but is a stoichiomorphism. E.g., for $s_{1},{\hat{r}}_{0}$: $\Sigma_{r\in {m_{R}}^{-1}\left({\hat{r}}_{0}\right)}\;\varphi \left(s_{1},r\right)=\varphi \left(s_{1},r_{0}\right)+\varphi \left(s_{1},r_{1}\right)=2\cdot k+0\cdot k=1\cdot \;2k=\;\varphi \left(m_{S}\left(s_{1}\right),{\hat{r}}_{0}\right)$.

This perspective justifies the non-standard bundling of reaction rates with stoichiometry in the definition of instantaneous stoichiometry *φ*, and the claim that morphisms defined over those bundles reveal properties of network ‘structure’. In fact, leaving rates out of the structure is mathematically problematic: it is easily possible to trade reaction rates with stoichiometry and obtain equivalent networks (consider *s*_0_ → ^2*k*^*s*_0_ + *s*_1_ vs. *s*_0_ → ^*k*^*s*_0_ + 2*s*_1_, and Figure 6(F)). Thus, rates and connectivity are sometimes interchangeable, and our definitions allow us to trade one with the other. It is possible to keep them separate, but at the cost of weaker mathematical results allowing for fewer emulation morphisms: our broader notion of network structure is simply more flexible and general. Most importantly, it still speaks about network properties that are state-independent and that can be determined by a purely syntactic examination of network presentation, such as the criteria for reactant morphisms and stoichiomorphisms.

As already mentioned, certain behavioral properties of individual networks, such as the number of steady states, may be characterized independently of reaction rates [1]. Here, however, we consider morphisms between two networks and their kinetic implications. It does not seem possible to assume that we can assign arbitrary rates to *both* networks and draw kinetic conclusions. But we can assign arbitrary rates to one network, and the rates of the other will follow in a systematic way (see the Change of Rates Theorem in Methods). This may be as rate-independent as we can hope for, when relating two networks.

## Conclusions

The main question that we study is: what are the conditions under which a network can emulate another one? We have shown relatively complex examples of emulation, and outlined technical answers that rely only on network structure. We also explained how our main question is of interest in the study of chemical and biological networks. The formal results tell us that network emulation relates the mass action kinetics of two networks, and that it can be characterized by morphisms defined only over network connectivity, stoichiometry, and rate constants. There are several ways in which we can interpret these results more informally.

Network morphisms that are emulations provide an *explanation* of network structure, in that they reveal structural connections between networks that entail kinetic connections. For example, we may suspect that the main purpose of a networks is to stabilize a system in one of two states. An emulation from that network to the AM network can confirm that suspicion, as a dynamical-system analysis could also reveal. Moreover, the mapping of reactions that entails emulation explains *how* stabilization is achieved mechanistically, and because of known results about the speed of AM convergence to steady state, *how fast* it can happen.

Network emulation can be understood in terms of *redundant implementation* of a particular network kinetics. Redundancy, in our examples, is not just simple replication: even when QI is exactly reproducing the kinetics of MI, it is doing so through an intricately interconnected set of reactions. Redundant implementation may seem to be wasteful, but there are situations in which it arises naturally. Biological networks, for example, are not known for their minimality. Redundancy there may be due to material constraints that prevent the minimal realization of a network but allow a more complex one. Redundancy also implies robustness: for identical kinetics, the more complex realization will be less perturbed by link or node deletion than the minimal realization (a precise characterization requires a theory of approximation). In these circumstances, criteria that allow us to check whether a network is capable of implementing another one may lead to a better understanding of the essential and inessential aspects of the more complex networks. For example, we can now understand which aspects of the cell cycle switches GW and CCR lead them to emulate AM exactly or approximately [16].

If we have reason to believe that a complex network is implementing a simpler one, we may also use this knowledge for *model reduction*. For example, we may know that concentrations of certain species follow similar trajectories, and therefore they may be identified. This resembles the notion of *abstraction* or *coarse-graining*, which has been widely studied for discrete systems as well as continuous ones [35,36]. Except that in that approach the abstract (simpler) network and the concrete (more complex) network should have, as much as possible, the same behavior for any parameter range. In our work, on the contrary, we seek an *emulation* or *fine-graining* of a simple network yielding a more complex network that retains the original behavior in appropriate conditions [37], but that may well diverge from it in general. Emulation is less constraining than abstraction in that it has to work only in specific contexts, namely in the connectivity context of the simpler network.

Another aspect of emulation is its *kinetic neutrality*. Neutral drift in RNA landscapes [38] allows RNA systems to explore alternative organizations, including more complex ones, without at first affecting functionality. Similarly, we can look at a sequence of emulations, such as the ones in Figure 3, as tracing (backwards) a neutral path in network space. If an evolutionary event accidentally produces a new network that, perhaps approximately, emulates the previous one, then the new network will not be immediately selected against. We show that the conditions for emulation need not be demanding, operating with the same parameters as the old network and providing for rate compensation. Emulation steps compose, so they can be repeated many times, resulting in very distant and possibly much more complex networks that can later evolve new functionality due to their extra degrees of freedom. Another possibility is a lateral jump: we can go first neutrally from AM to AMr, and then introduce another species to obtain CCR (which does not emulate AMr) while still emulating AM. Hence, CCR is reached in two gradual steps (one of which is not an emulation) instead of a single complex jump. The diagram in Figure 3, which is certainly not exhaustive, thus emphasizes the richness of network space: kinetic neutrality must be relatively rare, yet so many meaningful connections can be found.

In conclusion, we have shown that kinetic connections between networks can be established via network morphism defined only on state-independent attributes. In contrast, when studying directly the kinetic equations of a network, the structural properties are reduced to functional properties and disappear from sight, or at least become much more implicit. Therefore, network morphisms aid in understanding kinetic relationships between networks that are structural and not accidental: morphisms provide a structural *reason* for kinetic similarity.

## Methods

We define morphism between chemical reaction networks (CRNs) based on static network structure: connectivity, stoichiometry, and rate constants. These are therefore morphisms of the *syntax* of the networks; we then study implications about the kinetics. We prove two main theorems: an Emulation Theorem stating structural conditions under which two networks can have identical traces for any choice of initial conditions, and a Change of Rates Theorem generalizing that result to any choice or reaction rates as well. In Additional file 5 we discuss conditions under which the Emulation Theorem has an inverse.

Let *ℕ* be the natural numbers, *ℤ* be the integers, *ℝ* be the reals, *ℝ*_+_ be the non-negative reals, and *ℙ* be the strictly positive reals. A set *A* has cardinality |*A*|. We write *A* → *B* and *B*^*A*^ for the functions from *A* to *B*. When *f* ∈ *A* → *B* and *a* ∈ *A* we use *f*(*a*) and *f*_*a*_ for function application. A function *f* ∈ *A* → *B* has *images f*(*X* ⊆ *A*) = {*b* ∈ *B*| ∃ *a* ∈ *X f*(*a*) = *b*} and *fibers f*^- 1^(*b* ∈ *B*) = {*a* ∈ *A*|*f*(*a*) = *b*} (inverse images of singleton sets). A function *f* ∈ *A* × *B* → *ℝ* is a *matrix* of dimensions |*A*| × |*B*|, with matrix multiplication *f* · *g* and matrix transpose *f*^T^(*i*, *j*) = *f*(*j*, *i*).

### Chemical reaction networks (CRNs)

Let  be a universe of *species* and $S=\left\{s_{1},\ldots ,s_{n}\right\}\subseteq S$ a finite set. A *complex over S* is a function in *ℕ*^*S*^, representing the left hand side or right hand side of a reaction, associating to each species its multiplicity as a reactant or product. A *reaction r over S* is a triple (*ρ*,*π*,*k*) ∈ ℛ_*S*_ = *ℕ*^*S*^ × *ℕ*^*S*^ × ℙ, representing the reaction *ρ* → ^*k*^*π*. According to the standard chemical notation, we write:

(1) $$\rho \to^{k}\pi =\rho_{s_{1}}\cdot s_{1}+\cdots +\rho_{s_{n}}\cdot s_{n}\to^{k}\pi_{s_{1}}\cdot s_{1}+\cdots +\pi_{s_{n}}\cdot s_{n}$$

where *s*_*i*_ are the species, $\rho_{s_{i}}$ are the multiplicities (*stoichiometric numbers*) of the reactant species, $\pi_{s_{i}}$ are the multiplicities of the product species, and *k* is the reaction rate constant. We use 1^*st*^(*ρ*, *π*, *k*) = *ρ*.

The *net stoichiometry η*(*s*, *r*) of a species *s* in a reaction *r* = *ρ* → ^*k*^*π* is the difference between product and reactant multiplicity. The *(instantaneous) stoichiometry φ*(*s*, *r*) is *η*(*s*, *r*) multiplied by the rate constant:

$$\eta \left(s,\;r\right)=\eta \left(s,\rho \to^{k}\pi \right)=\pi_{s}-\rho_{s}\in \mathbb{Z}$$

$$\varphi \left(s,r\right)=\varphi \left(s,\rho \to^{k}\pi \right)=k\cdot \left(\pi_{s}-\rho_{s}\right)\in \mathbb{R}$$

A *Chemical Reaction Network (CRN)* is a pair (*S, R*) where *R* ⊆ ℛ_*S*_ is a finite set of reactions over *S*, such that:

$$\rho \to^{k}\pi ,\;\rho \to^{k'}\pi \in R\Rightarrow k=k'$$

(Two reactions *ρ* → ^*k*^*π*, *ρ* → ^*k* '^*π* are kinetically equivalent in mass action to a single reaction *ρ* → ^*k* + *k* '^*π*.)

As common [1,39,40], this definition of CRN considers only irreversible reactions, with reversible reactions modeled as pairs of opposite reaction. Moreover, it does not enforce conservation or detail balance laws so that it can cover the description of open chemical systems that, e.g., take energy or materials from the environment.

### Species maps and complex maps

Let (*S, R*) and $\left(\hat{S},\;\hat{R}\right)$ be two CRNs (we generally use ^ to indicate the target of a map). A *species map* between them is a map $m\in S\to \hat{S}$; we will use $m^{-1}\left(\hat{s}\right)=\left\{s\in S\left|m\left(s\right)=\hat{s}\right)\right\}$ for the *m*-fiber of $\hat{s}$. For any reaction $\rho \to^{k}\pi \in R\subseteq {ℛ}_{\hat{S}}$, like (1), we then have a reaction in ${ℛ}_{\hat{S}}$ that is intuitively its linear image via *m*:

$$\begin{array}{l} \rho_{s_{1}}\cdot m\left(s_{1}\right)+\cdots +\rho_{s_{n}}\cdot m\left(s_{n}\right)\to^{k}\;\pi_{s_{1}}\cdot m\left(s_{1}\right)+\cdots \\ \;+\pi_{s_{n}}\cdot m\left(s_{n}\right) \end{array}$$

But *m* is not necessarily injective, hence some of the species may be repeated in the image reaction, and the corresponding multiplicities must be summed to obtain appropriate $\hat{\rho },\;\hat{\pi }$ over $\hat{S}$. We can do this summing systematically by extending the species map $m\in S\to \hat{S}$ to a *complex map*$m\in {\mathbb{N}}^{S}\to {\mathbb{N}}^{\hat{S}}$ as follows, for any *σ* ∈ *ℕ*^*S*^ and $\hat{s}\in \hat{S}$ (note that $m{\left(\sigma \right)}_{\hat{s}}=0$ if $\hat{s}\notin m\left(S\right)$):

$$\begin{array}{ll} \;m{\left(\sigma \right)}_{\hat{s}} & =\sum_{s\in \left\{s\in S|m\left(s\right)=\hat{s}\right\}\;}\sigma_{s}\\ & =\sum_{s\in m^{-1}\left(\hat{s}\right)}\;\sigma_{s}\;\mathrm{where}\;m\left(\sigma \right)\in {\mathbb{N}}^{\hat{S}} \end{array}$$

Then the proper image of reaction (1) via *m* is *m*(*ρ*) → ^*k*^*m*(*π*), that is, for $\hat{S}=\left\{{\hat{s}}_{1},\ldots ,{\hat{s}}_{\hat{n}}\right\}$:

$$\begin{array}{ll} \;m\left(\rho \right)\to^{k}m\left(\pi \right) & =\;m{\left(\rho \right)}_{{\hat{s}}_{1}}\cdot {\hat{s}}_{1}+\ldots \\ & \;+m{\left(\rho \right)}_{{\hat{s}}_{\hat{n}}}\cdot {\hat{s}}_{\hat{n}}\;\to^{k}\;m{\left(\pi \right)}_{{\hat{s}}_{1}}\cdot {\hat{s}}_{1}+\ldots \\ & \;+m{\left(\pi \right)}_{{\hat{s}}_{\hat{n}}}\cdot {\hat{s}}_{\hat{n}} \end{array}$$

With these preliminaries, we can now define various morphisms between CRNs.

### Morphisms between CRNs

A *CRN morphism* from (*S, R*) to $\left(\hat{S},\;\hat{R}\right)$ is a pair of maps $m_{S}\in S\to \hat{S}$ and $m_{R}\in R\to \hat{R}$. We write $m\in \left(S,\;R\right)\to \left(\hat{S},\;\hat{R}\right)$ for $m=\left(m_{S},\;m_{R}\right)\in \left(S\to \hat{S}\right)\times \left(R\to \hat{R}\right)$, given that (*S, R*) and $\left(\hat{S},\;\hat{R}\right)$ are CRNs. See Figure 6 for some simple examples of morphisms between CRNs.

Linear algebra notation is sometimes useful for compactness. We write $m_{S}\in S\times \hat{S}\to \left\{0,\;1\right\}$ for the characteristic matrix of *m*_*S*_, such that $\forall s\in S\forall \hat{s}\in \hat{S}\;m_{S}\left(s,\;\hat{s}\right)=\left(\mathrm{if}\;m_{S}\left(s\right)=\hat{s}\;\mathrm{then}\;1\;\mathrm{else}\;0\right)$, and similarly for $m_{R}\in R\times \hat{R}\to \left\{0,\;1\right\}$. We write ***φ***_(*S*,*R*)_ ∈ *S* × *R* → *ℝ* for the *(instantaneous) stoichiometric matrix* such that ∀ *s* ∈ *S* ∀ *r* ∈ *R* ***φ***_(*S*,*R*)_(*s*, *r*) = *φ*(*s*, *r*). We write ***ρ***_(*S*, *R*)_ ∈ *S* × *R* → *ℕ* for the *reactant matrix* such that ∀ *s* ∈ *S* ∀ *ρ* → ^*k*^*π* ∈ *R* ***ρ***_(*S*, *R*)_(*s*, *ρ* → ^*k*^*π*) = *ρ*_*s*_.

A CRN morphism $m\in \left(S,\;R\right)\to \left(\hat{S},\;\hat{R}\right)$ is a *CRN homomorphism* if:

$$\forall \rho \to^{k}\pi \in R\;m_{R}\left(\rho \to^{k}\pi \right)=m_{S}\left(\rho \right)\to^{k}m_{S}\left(\pi \right)$$

that is, if the reaction map *m*_*R*_ is already determined by the species map *m*_*S*_ (extended to complexes). This tight relationship between the two networks implies also a relationship between their stoichiometry. A homomorphism does not preserve the stoichiometry of each species, but it preserves the sum stoichiometry of species in the same fiber, by the definition of *m*_*S*_ over complexes. We can say that a homomorphism *preserves stoichiometry over species fibers*, and this is expressed by the following easily derived property (see Additional file 5). Let $m\in \left(S,\;R\right)\to \left(\hat{S},\;\hat{R}\right)$ be a CRN homomorphism with ***φ*** = ***φ***_(*S*, *R*)_ and $\hat{\varphi }=\varphi_{\left(\hat{S},\;\hat{R}\right)}$, then:

$$\begin{array}{l} {m_{S}}^{T}\cdot \varphi =\hat{\varphi }\cdot {m_{R}}^{T}\;\mathrm{that}\;\mathrm{is}:\\ \forall \hat{s}\in \hat{S}\;\forall r\in R\;\sum_{s\in m^{-1}\left(\hat{s}\right)}\varphi \left(s,\;r\right)=\varphi \left(\hat{s},\;m\left(r\right)\right) \end{array}$$

(This property does not imply homomorphism, e.g., *m*(*s* → *s*) = 2*s* → 2*s*, or *m*(*s*_0_ → *s*_0_ + *s*_1_) = 2*s*_0_ → 2*s*_0_ + *s*_1_, determine maps between single-reaction CRNs that are not homomorphisms.)

Although homomorphisms produce the most natural examples of network morphisms, it is useful for our results to consider weaker morphisms that preserve just the reactant component of a reaction (which is helpful because the mass action of a reaction is defined on the reactants).

A CRN morphism $m\in \left(S,\;R\right)\to \left(\hat{S},\;\hat{R}\right)$ is a *CRN reactant morphism* if the reactant component of the reaction map *m*_*R*_ is already determined by the species map *m*_*S*_, that is:

$$\forall \rho \to^{k}\pi \in R\;\exists \hat{\pi },\;\hat{k}\;m_{R}\left(\rho \to^{k}\pi \right)=m_{S}\left(\rho \right)\to^{\hat{k}}\hat{\pi }$$

Then $m\in \left(S,\;R\right)\to \left(\hat{S},\;\hat{R}\right)$ is a CRN reactant morphism iff, with ***ρ*** = ***ρ***_(*S*, *R*)_ and $\hat{\rho }=\rho_{\left(\hat{S},\;\hat{R}\right)}$**:**

$$\begin{array}{l} {m_{S}}^{T}\cdot \rho =\hat{\rho }\cdot {m_{R}}^{T}\;\mathrm{that}\;\mathrm{is}:\\ \;\forall r\in R\;m_{S}\left(1^{\mathrm{st}}\left(r\right)\right)=1^{\mathrm{st}}\left(m_{R}\left(r\right)\right) \end{array}$$

In the sequel we often omit the subscripts on *m*_*S*_ and *m*_*R*_.

The following property is complementary to the homomorphism property and is central to all that follows. A *CNR stoichiomorphism* is a CRN morphism $m\in \left(S,\;R\right)\to \left(\hat{S},\;\hat{R}\right)$ that satisfies:

$$\begin{array}{l} \varphi \cdot m_{R}=m_{S}\cdot \hat{\varphi }\;\mathrm{that}\;\mathrm{is}:\\ \forall s\in S\;\forall \hat{r}\in \hat{R}\;\sum_{r\in m^{-1}\left(\hat{r}\right)}\;\varphi \left(s,\;r\right)=\varphi \left(m\left(s\right),\;\hat{r}\right) \end{array}$$

We can say that a stoichiomorphism *preserves stoichiometry over reaction fibers*, meaning that the sum of the stoichiometry of any *s* ∈ *S* in the reactions that map to any $\hat{r}\in \hat{R}$, must equal the stoichiometry of *m*(*s*) in $\hat{r}$.

If a stoichiomorphism is also a homomorphism, we obtain from above that $m_{S}\cdot {m_{S}}^{T}\cdot \varphi =m_{S}\cdot \hat{\varphi }\cdot {m_{R}}^{T}=\varphi \cdot m_{R}\cdot {m_{R}}^{T}$, that is:

$$\begin{array}{l} \forall s\in S\;\forall r\in R\;\sum_{s^{\prime }\in m^{-1}\left(m\left(s\right)\right)}\varphi \left(s^{\prime },\;r\right)=\varphi \left(m\left(s\right),\;m\left(r\right)\right)\\ \;=\sum_{r^{\prime }\in m^{-1}\left(m\left(r\right)\right)}\varphi \left(s,r^{\prime }\right) \end{array}$$

If *m* is injective and surjective then the transposes are inverses, hence both the homomorphism and stoichiomorphism properties reduce to $\varphi =m_{S}\cdot \hat{\varphi }\cdot {m_{R}}^{T}$, that is to the natural property:

$$\forall s\in S\;\forall r\in R\;\varphi \left(s,\;r\right)=\varphi \left(m\left(s\right),\;m\left(r\right)\right)$$

The combination of homomorphism and stoichiomorphism forms a natural notion of network correspondence, strongly preserving both the graph structure and the stoichiometric structure of a network, while not requiring in general injectivity or surjectivity of the mapping. Many typical examples fall into this combined class. However, weaker notions of network morphisms are also useful, so we investigate stoichiomorphisms separately from homomorphisms, and in particular we combine them with reactant morphisms.

### Remark – homomorphic projection

Given a CRN (*S, R*) and a species map $m_{S}\in S\to S$, the *homomorphic projection* of (*S, R*) via *m*_*S*_ is the CRN (*m*_*S*_(*S*), *m*_*R*_(*R*)) where *m*_*R*_(*ρ* → ^*k*^*π*) = *m*_*S*_(*ρ*) → ^*k*^*m*_*S*_(*π*). By construction (*m*_*S*_(*S*), *m*_*R*_(*R*)) is a CRN, because *R* ⊆ ℛ_*S*_ and *ρ* → ^*k*^*π* ∈ *R* imply *ρ* ∈ *ℕ*^*S*^ and $m_{S}\left(\rho \right)\in {\mathbb{N}}^{m_{S}\left(S\right)}$, and similarly for *π*, hence $m_{ℛ}\left(\rho \to^{k}\pi \right)\in {{ℛ}_{m_{S}}}_{\left(S\right)}$ and $m_{R}\left(R\right)\subseteq {ℛ}_{m_{S}\left(S\right)}$. Moreover, the homomorphic projection is a CRN epimorphism: *m*_*S*_ and *m*_*R*_ are surjective. So if ρ^→k^π^,ρ^→k^'π^∈mRR then there is some *ρ* → ^*k*^*π*, *ρ* → ^*k* '^*π* ∈ *R* with $\hat{\rho }=m_{S}\left(\rho \right)$, $\hat{\pi }=m_{S}\left(\pi \right)$, $\hat{k}=k$, k^'=k'. Since (*S, R*) is a CRN we have *k* = *k*’ and hence k^=k^'.

### Remark – mathematical and graphical representations of CRNs

Following [40], a CRN is defined above as a finite set of species *S* ⊆ *S* and a reaction relation *R* ⊆ ℛ_*S*_ = *ℕ*^*S*^ × *ℕ*^*S*^ × *ℙ* over complexes *ℕ*^*S*^. In the CRNT approach [1], a CRN is instead defined as finite set of complexes *C* ⊆ *ℕ*^*S*^ and a reaction relation *R* ⊆ ℛ_*C*_ = *C* × *C* × *ℙ*. We can of course translate between these two representations: our reaction triples *ρ* → ^*k*^*π* = (*ρ*, *π*, *k*) are already in the CRNT format. We even obtain the same graphical representation by drawing the directed graphs of the relations ℛ_*S*_ and ℛ_*C*_ with *complexes as nodes* and *reactions as edges* (labeling the edges with the rate *ℙ*), which is the most common depiction of CRNs.

The morphisms that arise from those graphs, however, are different from ours. In the CRNT case we would naturally map *complexes to complexes*, but it is hard to see what kinetic properties could be preserved by such mappings, unless we required further relationships between the complexes. Our morphisms instead map *species to species*, which constrains the relationships between the complexes being mapped. The notion of morphism that we study is therefore predicated on the (*S, R*) representation.

To better visualize these CRNs and their morphisms, we draw graphs where nodes represent species, not complexes. A reaction then becomes a ‘directed multi-edge’ with sets of species as source and target. A good way to visualize such edges is as Petri nets: a CRN is drawn as a directed bipartite graph between (round) species nodes and (square) reaction nodes (many bipartite variants exist [9], but we conform to Petri nets [34]). A species that occurs as a reactant in a reaction is connected to it by a directed edge, with as many such edges as the stoichiometric number of the species. A reaction that produces a species as a product is connected to it by a directed edge, with as many such edges as the stoichiometric number of the species. If we omit an arrowhead on an edge, the edge is by convention directed from the species node to the reaction node. Reaction rates are affixed to the reaction nodes; if omitted they are equal to 1. A CRN morphism is then represented as a mapping between species nodes and a mapping between reaction nodes, between two Petri nets (Figure 6).

### CRN kinetics

We now give a formulation of standard mass action kinetics, for the purpose of next presenting results about the connection between CRN morphisms and kinetics.

A species *s* has dimension mol (*amount of substance*); its concentration has dimension *molarity* M = mol · l^‒ 1^. A reaction *r* = *ρ* → ^*k*^*π* has *order* |*r*| = ∑ _*s* ∈ *S*_*ρ*_*s*_ and its rate *k* has dimension s^‒ 1^ · M^1 ‒ |*r*|^.

A *state*$v\in {\mathbb{R}}_{+}^{S}$ of a CRN (*S*, *R*) is a vector of concentrations for each species, of dimension M^*S*^. For a reaction *r* ∈ *R* over *S*, its *mass action*$\left[r\right]\in {\mathbb{R}}_{+}^{S}\to {\mathbb{R}}_{+}$ of dimension M^*S*^ → M^|*r*|^ is the product of the reagent concentrations according to their multiplicity (the term ***v***^*ρ*^ is just an abbreviation for the product below [1]):

$${\left[r\right]}_{v}={\left[\rho \to^{k}\pi \right]}_{v}=\prod_{s\in S}v_{s}^{\rho_{s}}=v^{\rho }$$

The *(autonomous) differential system* of a CRN (*S*, *R*) is the following map $F\in {\mathbb{R}}_{+}^{S}\to {\mathbb{R}}^{S}$ of dimension M^*S*^ → (M · s^- 1^)^*s*^. For each state ***v*** and species *s*, it is the sum for all reactions *r* of the stoichiometry of the species *s* in the reaction *r* multiplied by the mass action of the reaction *r* in the state ***v***:

$$F\left(v\right)\left(s\right)=\sum_{r\in R}\varphi \left(s,\;r\right)\cdot {\left[r\right]}_{v}$$

where *F*(***v***)(*s*) represents the instantaneous change of concentration (derivative) of *s* in state ***v***. *F* is commonly presented as a coupled system of Ordinary Differential Equations for each *s* ∈ *S*, integrated over time *t*:

$$\frac{dv_{s}}{\mathrm{dt}}=F\left(v\right)\left(s\right)=\sum_{\rho \to^{k}\pi \in R}k\cdot \left(\pi_{s}-\rho_{s}\right)\cdot \prod_{s^{\prime }\in S}{v_{s^{\prime }}}^{\rho_{s^{\prime }}}$$

For each initial state ***v*** the differential system *F* has a unique maximal (for some *t* ≤ + ∞) differentiable *solution*$f\in \left[0,\;\left(t\right)\to {\mathbb{R}}_{+}^{S}\right)$ such that *f*(0) = ***v*** and $\dot{f}=F\circ f$, where $\dot{f}$ is the (time) derivative of *f* [Cauchy-Lipschitz]. Since *F* arises from a CRN, the solution is everywhere non-negative [1].

Note that it is traditional to group the three factors of the differential system as (*π*_*s*_ - *ρ*_*s*_) · (*k* · ***v***^*ρ*^), where *k* · ***v***^*ρ*^ are the *rate functions*, which are interpreted as the *kinetics* of the system: kinetics other than mass action can be investigated by varying the rate functions. In mass action, however, the grouping (*k* · (*π*_*s*_ - *ρ*_*s*_)) · ***v***^*ρ*^ is also natural because *k* · (*π*_*s*_ - *ρ*_*s*_) = *φ*(*s*, *r*) is the state-independent factor and ***v***^*ρ*^ = [*r*]_***v***_ is the state-dependent factor, so that the *syntactic* structure of the network is gathered in *φ*(*s*, *r*). By representing reactions as triples (*ρ*, *π*, *k*) we have already committed to a rate function that depends on a single rate parameter *k*. Other common kinetics can be approximated in mass action, such as Michaelis-Menten enzyme kinetics, and Hill kinetics as in the triplet motif of Figure 2.

### CRN emulation

We can now state the key kinetic property that is captured via stoichiomorphisms. A map $m\in \left(S,\;R\right)\to \left(\hat{S},\;\hat{R}\right)$ is a *CRN emulation* if the following holds for the respective differential systems $F,\;\hat{F}$, where *F* emulates $\hat{F}$:

$$\forall \hat{v}\in {\mathbb{R}}_{+}^{\hat{S}}\;F\left(\hat{v}\circ m\right)=\hat{F}\left(\hat{v}\right)\circ m$$

That means that $\forall s\in S\;F\left(\hat{v}\circ m\right)\left(s\right)=\hat{F}\left(\hat{v}\right)\left(m\left(s\right)\right)$, stating that the derivative of *s* in state $\hat{v}\circ m$ is equal to the derivative of *m(s)* in state $\hat{v}$, for any state $\hat{v}$. As a commuting diagram, even though $m\in S\to \hat{S}$, we have that $-\circ m\in {\mathbb{R}}^{\hat{S}}\to {\mathbb{R}}^{S}$, and hence the arrows involving *m* are reversed:

$$\begin{array}{ccccccccc} & & & & F & & & & \\ & & & {\mathbb{R}}^{S} & \to & {\mathbb{R}}^{S} & & & \\ - & \circ & m & ↑ & & ↑ & - & \circ & m\\ & & & {\mathbb{R}}^{\hat{S}} & \hat{F} & {\mathbb{R}}^{\hat{S}} & & & \\ & & & & \to & & & & \end{array}$$

Starting from the bottom left towards the top right, if we apply the emulating *F* to a state $\hat{v}\circ m$ of *S* which is a copy under *m*^-1^ of a state $\hat{v}$ of $\hat{S}$, we should obtain derivatives that are copies under *m*^-1^ of the derivatives of the emulated $\hat{F}$ in state $\hat{v}$.

That ‘copying’ of derivatives extends to whole trajectories. Suppose *m* is such a CNR emulation, and $f,\;\hat{f}$ are the solutions of $F,\;\hat{F}$ for initial states $\hat{v}\circ m,\;\hat{v}$ respectively. We then have that for all *s* ∈ *S*:

$$\begin{array}{l} f\left(0\right)\left(s\right)={\left(\hat{v}\circ m\right)}_{s}={\hat{v}}_{m\left(s\right)}=\hat{f}\left(0\right)\left(m\left(s\right)\right)\\ \dot{f}\left(0\right)\left(s\right)=\left(F\circ f\right)\left(0\right)\left(s\right)=F\left(f\left(0\right)\right)\left(s\right)=F\left(\hat{v}\circ m\right)\left(s\right)=\\ \left(\hat{F}\left(\hat{v}\right)\circ m\right)\left(s\right)=\hat{F}\left(\hat{f}\left(0\right)\right)\left(m\left(s\right)\right)=\left(\hat{F}\circ \hat{f}\right)\left(0\right)\left(m\left(s\right)\right)=\\ \;\dot{\hat{f}}\left(0\right)\left(m\left(s\right)\right) \end{array}$$

Hence both $f,\;\hat{f}$ and $\dot{f},\;\dot{\hat{f}}$ coincide at 0, and therefore every *s*-trajectory of *F* will coincide with the *m*(*s*) -trajectory of $\hat{F}$ until the maximal time of $\hat{f}$. (If *m* is not surjective, then $\hat{f}$ as a whole could stop sooner than *f*, e.g., if $\hat{S}$ has an extra species *s*′ and $\hat{R}$ has an extra reaction 2*s*′ → 3*s*′).

In summary, an emulation $m\in \left(S,\;R\right)\to \left(\hat{S},\;\hat{R}\right)$ is such that for *any* initial condition $\hat{v}$ for $\left(\hat{S},\;\hat{R}\right)$ there is *some* initial condition for (*S, R*), namely $\hat{v}\circ m$, such that (*S, R*) can exactly reproduce the kinetics of $\left(\hat{S},\;\hat{R}\right)$.

### Emulation theorem

We can now relate CRN stoichiomorphisms to CRN emulations.

### Lemma - mass action

1) Let $m\in \left(S,\;R\right)\to \left(\hat{S},\;\hat{R}\right)$ be any CRN morphism. For any state $\hat{v}\in {ℛ}_{+}^{\hat{S}}$ and complex *ρ* ∈ *ℕ*^*S*^, we have ${\left(\hat{v}\circ m\right)}^{\rho }={\hat{v}}^{m\left(\rho \right)}$.

2) Let $m\in \left(S,\;R\right)\to \left(\hat{S},\;\hat{R}\right)$ be a CRN reactant morphism. For any reaction *r* ∈ *R* and state $\hat{v}\in {\mathbb{R}}_{+}^{\hat{S}}$, we have ${\left[r\right]}_{\hat{v}\circ m}={\left[m\left(r\right)\right]}_{\hat{v}}$.

Proof

1) By the Fiber Lemma (see Additional file 5), for any function $m\in S\to \hat{S}$ with $g\in S\times \hat{S}\to {\mathbb{R}}_{+}$ we have $\prod_{\hat{s}\in \hat{S}}\prod_{s\in m^{-1}\left(\hat{s}\right)}g\left(s,\;\hat{s}\right)=\prod_{s\in S}g\left(s,m\left(s\right)\;\right)$. Then:

$$\begin{array}{ll} {\hat{v}}^{m\left(\rho \right)}=\prod_{\hat{s}\in \hat{S}}{\hat{v}}_{\hat{s}}^{m{\left(\rho \right)}_{\hat{s}}} & \mathrm{notational}\;\mathrm{definition}\\ =\prod_{\hat{s}\in \hat{S}}{\hat{v}}_{\hat{S}}^{\sum_{s\in m^{-1}\left(\hat{s}\right)}\rho_{s}} & \mathrm{by}\;\mathrm{definition}\;\mathrm{of}\;m\left(\rho \right)\\ =\prod_{\hat{s}\in \hat{S}}\prod_{s\in m^{-1}\left(\hat{s}\right)}{\hat{v}}_{\hat{s}}^{\;\rho_{s}} & \mathrm{by}\;\mathrm{distribution}\;\mathrm{of}\;\mathrm{exponents}\\ =\prod_{s\in S}{{\hat{v}}_{m\left(s\right)}}^{\rho_{s}} & \mathrm{by}\;\mathrm{the}\;\mathrm{Fiber}\;\mathrm{Lemma}\;\mathrm{with}\;g\left(x,\;y\right)\;=\;{{\hat{v}}_{y}}^{\rho_{x}}\\ ={\prod_{s\in S}\left(\hat{v}\circ m\right)}_{s}^{\rho_{s}} & \mathrm{by}\;\mathrm{definition}\;\mathrm{of}\;\circ \\ ={\left(\hat{v}\circ m\right)}^{\rho } & \mathrm{notational}\;\mathrm{definition} \end{array}$$

2) Let *r* = *ρ* → ^*k*^*π*. Then:

$$\begin{array}{ll} {\left[m\left(r\right)\right]}_{\hat{v}}={\left[m\left(\rho \right)\to^{\hat{k}}\hat{\pi }\right]}_{\hat{v}} & \;\mathrm{by}\;\mathrm{definition}\;\mathrm{of}\;\mathrm{reactant}\;\mathrm{morphism}\;\mathrm{for}\;\mathrm{some}\;\hat{\pi },\;\hat{k}\\ ={\hat{v}}^{m\left(\rho \right)} & \;\mathrm{by}\;\mathrm{definition}\;\mathrm{of}\;\mathrm{mass}\;\mathrm{action}\;\mathrm{of}\;a\;\mathrm{reaction}\\ ={\left(\hat{v}\circ m\right)}^{\rho } & \;\mathrm{by}\;\left(1\right)\\ ={\left[\rho \to^{k}\pi \right]}_{\hat{v}\circ m}={\left[r\right]}_{\hat{v}\circ m} & \;\mathrm{by}\;\mathrm{definition}\;\mathrm{of}\;\mathrm{mass}\;\mathrm{action}\;\mathrm{of}\;a\;\mathrm{reaction} \end{array}$$

■

### Theorem - emulation

If $m\in \left(S,\;R\right)\to \left(\hat{S},\;\hat{R}\right)$ is a CRN reactant morphism and a CRN stoichiomorphism, then it is a CNR emulation; that is, for any $\hat{v}\in {\mathbb{R}}_{+}^{\hat{S}}$ the differential systems *F* of (*S*, *R*) and $\hat{F}$ of $\left(\hat{S},\;\hat{R}\right)$ commute via *m*:

$$F\left(\hat{v}\circ m\right)=\hat{F}\left(\hat{v}\right)\circ m$$

Proof

We need to show, by definition of differential systems *F* and $\hat{F}$, that ∀ *s* ∈ *S*:

$$\sum_{r\in R}\varphi \left(s,\;r\right)\cdot {\left[r\right]}_{\hat{v}\circ m}=\sum_{\hat{r}\in \hat{R}}\varphi \left(m\left(s\right),\hat{r}\right)\cdot {\left[\hat{r}\right]}_{\hat{v}}$$

Since *m* is a stoichiomorphism, we have that $\forall s\in S,\forall \hat{r}\in \hat{R}$:

$$\sum_{r\in m^{-1}\left(\hat{r}\right)}\varphi \left(s,r\right)=\varphi \left(m\left(s\right),\;\hat{r}\right)$$

We multiply both sides by ${\left[\hat{r}\right]}_{\hat{v}}$, distribute, and sum over all $\hat{r}\in \hat{R}$, obtaining the desired right hand side:

$${\sum_{\hat{r}\in \hat{R}}\sum_{r\in m^{-1}\left(\hat{r}\right)}\varphi \left(s,r\right)\cdot \left[\hat{r}\right]}_{\hat{v}}=\sum_{\hat{r}\in \hat{R}}\varphi \left(m\left(s\right),\hat{r}\right)\cdot {\left[\hat{r}\right]}_{\hat{v}}$$

For the left hand side, by the Fiber Lemma, for any function $m\in R\to \hat{R}$ with $g\in R\times \hat{R}\to \mathbb{R}$ we have:

$$\sum_{\hat{r}\in \hat{R}}\sum_{r\in m^{-1}\left(\hat{r}\right)}g\left(r,\hat{r}\right)=\sum_{r\in R}g\left(r,m\left(r\right)\right)$$

For $g\left(x,y\right)=\varphi \left(s,x\right)\cdot {\left[y\right]}_{\hat{v}}$ we obtain:

$${\sum_{\hat{r}\in \hat{R}}\sum_{r\in m^{-1}\left(\hat{r}\right)}\varphi \left(s,r\right)\cdot \left[\hat{r}\right]}_{\hat{v}}=\sum_{r\in R}\varphi \left(s,r\right)\cdot {\left[m\left(r\right)\right]}_{\hat{v}}$$

Since *m* is a reactant morphism, by the Mass Action Lemma, $\forall r\in R{\left[m\left(r\right)\right]}_{\hat{v}}={\left[r\right]}_{\hat{v}\circ m}$, hence we have the desired left hand side:

$$\sum_{r\in R}\varphi \left(s,r\right)\cdot {\left[m\left(r\right)\right]}_{\hat{v}}=\sum_{r\in R}\varphi \left(s,r\right)\cdot {\left[r\right]}_{\hat{v}\circ m}$$

■

Note that a stoichiomorphism need not be a homomorphism for the theorem to hold: both rates and products may be allowed to vary as long as the stoichiomorphism property is satisfied. However the stoichiomorphism needs to be a reactant morphism because that is the basis of the Mass Action lemma. Because of the form of the differential system *F*(***v***)(*s*) = ∑ _*r* ∈ *R*_*φ*(*s*, *r*) · [*r*]_***v***_, the stoichiomorphism condition supports the *φ*(*s*, *r*) factor, while the reactant morphism condition supports the [*r*]_***v***_ factor. As matrix equations, the assumptions for this theorem are ${m_{S}}^{T}\cdot \rho =\hat{\rho }\cdot {m_{R}}^{T}$ (reactant morphism) and $\varphi \cdot m_{R}=m_{S}\cdot \hat{\varphi }$ (stoichiomorphism). (See the supporting examples 10, 11, 12, in Additional file 2.)

To conclude this section, the following proposition shows that any emulation, and hence any morphism that is both a reactant morphism and a stoichiomorphism, preserves steady states from the target CRN to the source CRN. Therefore the existence of a stoichiomorphism/reactant morphism can be used to determine, syntactically, that a CRN has at least certain steady states inherited from another CRN whose steady states are known.

### Proposition – steady states

Let $m\in \left(S,\;R\right)\to \left(\hat{S},\;\hat{R}\right)$ be a CRN emulation, and $F,\hat{F}$ be the differential systems of $\left(S,R\right),\;\left(\hat{S},\hat{R}\right)$. Any steady state of $\hat{F}$ under *m* is a steady state of *F*, that is:

$$\forall \hat{v}\in {\mathbb{R}}_{+}^{\hat{S}}\left(\forall s\in S\;\hat{F}\left(\hat{v}\right)\left(m\left(s\right)\right)=0\right)\Rightarrow \left(\forall s\in S\;F\left(\hat{v}\circ m\right)\left(s\right)=0\right)$$

Conversely, if *m* is also a bijection on species, then any steady state of *F* is a steady state of $\hat{F}$ under *m*:

$$\forall v\in {\mathbb{R}}_{+}^{S}\left(\forall s\in S\;F\left(v\right)\left(s\right)=0\right)\Rightarrow \left(\forall s\in S\;\hat{F}\left(v\circ m^{-1}\right)\left(m\left(s\right)\right)=0\right)$$

Proof

We have that $\forall \hat{v}\in {\mathbb{R}}_{+}^{\hat{S}}\forall s\in S\;\hat{F}\left(\hat{v}\right)\left(m\left(s\right)\right)=F\left(\hat{v}\circ m\right)\left(s\right)$.

Hence, if $\hat{v}\in {\mathbb{R}}_{+}^{\hat{S}}$ is a (partial) steady state of $\left(\hat{S},\;\hat{R}\right)$ via *m*, that is if $\forall s\in S\;\hat{F}\left(\hat{v}\right)\left(m\left(s\right)\right)=0$, then $\forall s\in S\;F\left(\hat{v}\circ m\right)\left(s\right)=0$, so $\hat{v}\circ m\in {\mathbb{R}}_{+}^{S}$ is a (full) steady state of (*S, R*). The same holds if $\hat{v}$ is a full steady state.

Conversely, if *m* is a bijection on species and $v\in {\mathbb{R}}_{+}^{S}$ is a steady state of *F*, then take $\hat{v}=v\circ m^{‒1}$. We have that for all *s* ∈ *S*, $\hat{F}\left(\hat{v}\right)\left(m\left(s\right)\right)=F\left(\hat{v}\circ m\right)\left(s\right)=F\left(v\right)\left(s\right)=0$; hence $\hat{v}$ is a steady state of $\hat{F}$.

■

The converse direction fails if *m* is not injective: if ***v*** is any steady state of (*S, R*), then there might not be any $\hat{v}$ with $\hat{v}\circ m=v$ (if ‘all *v*_*S*_ differ’) and hence no corresponding steady state in $\left(\hat{S},\;\hat{R}\right)$.

### Change of rates theorem

We now generalize the Emulation Theorem, which allows us to choose arbitrary initial conditions, to also allow choosing arbitrary rates for the target CRN. (See the supporting Example 10 in Additional file 2.)

A *change of rates* is a CRN morphism *ι* ∈ (*S*, *R*) → (*S*, *R*′) such that *ι*_*S*_ ∈ *S* → *S* is the identity and *ι*_*R*_ ∈ *R* → *R*′ is a bijection such that: $\iota_{R}\left(\rho \to^{k}\pi \right)=\rho^{\prime }\to^{k^{\prime }}\pi^{\prime }\Rightarrow \rho =\rho^{\prime }\&\pi =\pi^{\prime }$. Then *ι*^- 1^ ∈ (*S*, *R*′) → (*S*, *R*) is also a change of rates. As usual, we omit the subscripts on *ι*_*S*_, *ι*_*R*_.

### Theorem - change of rates

Let $m\in \left(S,R\right)\to \left(\hat{S},\hat{R}\right)$ be a stoichiomorphism, and $\hat{\iota }\in \left(\hat{S},\hat{R}\right)\to \left(\hat{S},{\hat{R}}^{\prime }\right)$ be any change of rates. Then there is a change of rates $\iota \in \left(S,R\right)\to \left(S,R^{\prime }\right)$ such that $\hat{\iota }\circ m\circ \iota^{-1}\in \left(S,R^{\prime }\right)\to \left(\hat{S},{\hat{R}}^{\prime }\right)$ is a stoichiomorphism.

Proof

**Figure 7:**
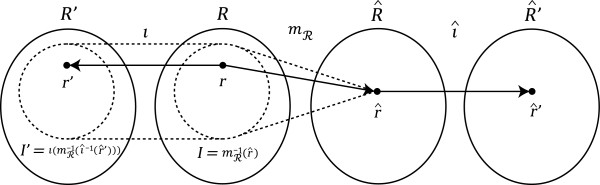
Morphisms in the change of rates theorem.

Take *ι* ∈ (*S*, *R*) → (*S*, *R*′) to be the change of rates such that (see Figure 7):

$$\begin{array}{l} \forall r=\rho \to^{k}\pi \in R\;\forall \hat{r}=\hat{\rho }\to^{\hat{k}}\hat{\pi }\in \hat{R}\;\forall {\hat{r}}^{\prime }\\ \;={\hat{\rho }}^{\prime }\to^{{\hat{k}}^{\prime }}{\hat{\pi }}^{\prime }\in {\hat{R}}^{\prime }\\ m\left(r\right)=\hat{r}\;\&\;\hat{\iota }\left(\hat{r}\right)={\hat{r}}^{\prime }\Rightarrow \;\iota \left(r\right)\\ \;=\rho \to^{k\cdot \frac{{\hat{k}}^{\prime }}{\hat{k}}}\pi \end{array}$$

Note that without the condition on CRNs that $\rho \to^{k_{1}}\pi ,\;\rho \to^{k_{2}}\pi \in R\Rightarrow \;k_{1}=k_{2}$, we might have that *ι* is not injective, if we had two such reactions in *R* with *k*_1_ ≠ *k*_2_, and for some values $k_{1}\cdot \frac{{\hat{k}}_{1}^{\prime }}{{\hat{k}}_{1}}=k_{2}\cdot \frac{{\hat{k}}_{2}^{\prime }}{{\hat{k}}_{2}}$. This is the one place where we take advantage of that condition, so that *ι* is injective and hence is a change of rates.

Take any *s* ∈ *S* and any ${\hat{r}}^{\prime }={\hat{\rho }}^{\prime }\to^{{\hat{k}}^{\prime }}{\hat{\pi }}^{\prime }\in {\hat{R}}^{\prime }$.

Let $\hat{r}=\hat{\rho }\to^{\hat{k}}\hat{\pi }={\hat{\iota }}^{-1}\left({\hat{r}}^{\prime }\right)$, implying $\hat{\rho }={\hat{\rho }}^{\prime }\;\&\;\hat{\pi }={\hat{\pi }}^{\prime }$ because $\hat{\iota }$ is a change of rates.

Let $r=\rho \to^{k}\pi \in I=m^{-1}\left(\hat{r}\right)\subseteq R$, and let $\iota \left(r\right)=r^{\prime }=\rho^{\prime }\to^{k^{\prime }}\pi^{\prime }\in I^{\prime }=\iota \left(m^{-1}\left({\hat{\iota }}^{-1}\left(\hat{r^{\prime }}\right)\right)\right)\subseteq R^{\prime }$. By construction of *ι* we have that *ρ*′ = *ρ*, *π*′ = *π*, and $k^{\prime }=k\cdot \frac{{\hat{k}}^{\prime }}{\hat{k}}$. Hence $k\cdot \left(\pi_{s}-\rho_{s}\right)=k^{\prime }\cdot \frac{\hat{k}}{{\hat{k}}^{\prime }}\cdot \left(\pi_{s}^{\prime }-\rho_{s}^{\prime }\right)$. Since *I* is in bijection with *I*′, we then have:

$$\sum_{\rho \to^{k}\pi \in I}k\cdot \left(\pi_{s}-\rho_{s}\right)=\sum_{\rho^{\prime }\to^{k^{\prime }}\pi^{\prime }\in I^{\prime }}k^{\prime }\cdot \frac{\hat{k}}{{\hat{k}}^{\prime }}\cdot \left(\pi_{s}^{\prime }-\rho_{s}^{\prime }\right)$$

where on the right hand side $\hat{k}$ and ${\hat{k}}^{\prime }$ are fixed for the whole sum by the initial choice of $\hat{r^{\prime }}$ and $\hat{r}={\hat{\iota }}^{-1}\left({\hat{r}}^{\prime }\right)$. Since *m* is a stoichiomorphism, we have, for the chosen *s* and $\hat{r}$:

$$\sum_{\rho \to^{k}\pi \in I}k\cdot \left(\pi_{s}-\rho_{s}\right)=\hat{k}\cdot \left({\hat{\pi }}_{m\left(s\right)}-{\hat{\rho }}_{m\left(s\right)}\right)$$

From the previous equality, using also on the right the fact that $\hat{\rho }={\hat{\rho }}^{\prime }$ and $\hat{\pi }={\hat{\pi }}^{\prime }$:

$$\sum_{\rho^{\prime }\to^{k^{\prime }}\pi^{\prime }\in I^{\prime }}k^{\prime }\cdot \frac{\hat{k}}{{\hat{k}}^{\prime }}\cdot \left(\pi_{s}^{\prime }-\rho_{s}^{\prime }\right)=\hat{k}\cdot \left({\hat{\pi }}_{m\left(s\right)}^{\prime }-{\hat{\rho }}_{m\left(s\right)}^{\prime }\right)$$

Multiplying both sides by $\frac{\hat{k^{\prime }}}{\hat{k}}$ and distributing on the left, and then discharging the initial quantification:

$$\begin{array}{l} \forall s\in S\forall {\hat{\rho }}^{\prime }\to^{{\hat{k}}^{\prime }}{\hat{\pi }}^{\prime }\in {\hat{R}}^{\prime }\\ \;\sum_{\rho^{\prime }\to^{k^{\prime }}\pi^{\prime }\in I^{\prime }}k^{\prime }\cdot \left(\pi_{s}^{\prime }-\rho_{s}^{\prime }\right)=\hat{k^{\prime }}\cdot \left({\hat{\pi^{\prime }}}_{m\left(s\right)}-{\hat{\rho^{\prime }}}_{m\left(s\right)}\right) \end{array}$$

That is, since $m\left(s\right)=\left(\hat{\iota }\circ m\circ \iota^{-1}\right)\left(s\right)$ and $I^{\prime }=\iota \left(m^{-1}\left({\hat{\iota }}^{-1}\left(\hat{r^{\prime }}\right)\right)\right)={\left(\hat{\iota }\circ m\circ \iota^{-1}\right)}^{-1}\left(\hat{r^{\prime }}\right)$:

$$\begin{array}{l} \forall s\in S\;\forall \hat{r^{\prime }}\in \hat{R^{\prime }}\;\sum_{r^{\prime }\in {\left(\hat{\iota }\circ m\circ \iota^{-1}\right)}^{-1}\left(\hat{r^{\prime }}\right)}\varphi \left(s,r^{\prime }\right)\\ \;=\varphi \left(\left(\hat{\iota }\circ m\circ \iota^{-1}\right)\left(s\right),\hat{r^{\prime }}\right) \end{array}$$

Which means that $\hat{\iota }\circ m\circ \iota^{-1}$ is a stoichiomorphism.

■

### Corollary - emulation under reactant morphism

If $m\in \left(S,R\right)\to \left(\hat{S},\hat{R}\right)$ is a CRN reactant morphism and a CRN stoichiomorphism, then for *any* change of rates $\hat{\iota }\in \left(\hat{S},\hat{R}\right)\to \left(\hat{S},{\hat{R}}^{\prime }\right)$, there is *some* change of rates *ι* ∈ (*S*, *R*) → (*S*, *R*′) such that $\hat{\iota }\circ m\circ \iota^{-1}\in \left(S,R^{\prime }\right)\to \left(\hat{S},\hat{R^{\prime }}\right)$ is a CRN reactant morphism and a CRN emulation.

Proof

By the Change of Rates Theorem for any $\hat{\iota }$ there is a *ι* such that $\hat{\iota }\circ m\circ \iota^{-1}$ is a stoichiomorphism. Moreover, if *m* is a reactant morphism then $\hat{\iota }\circ m\circ \iota^{-1}$ is too, because $\hat{\iota }$, *ι* are changes of rates and so: $\left(\hat{\iota }\circ m\circ \iota^{-1}\right)\left(\rho \to^{k^{\prime }}\pi \right)=\left(\hat{\iota }\circ m\right)\left(\rho \to^{k}\pi \right)=\hat{\iota }\left(m\left(\rho \right)\to^{\hat{k}}\hat{\pi }\right)=m\left(\rho \right)\to^{\hat{k^{\prime }}}\hat{\pi }$, where $m\left(\rho \right)=\left(id_{\hat{S}}\circ m\circ id_{S}\right)\left(\rho \right)=\left(i\circ m\circ \iota^{-1}\right)\left(\rho \right)$ because $\hat{\iota }$, *ι* are identities on species. Therefore, by the Emulation Theorem, $\hat{\iota }\circ m\circ \iota^{-1}$ is an emulation.

■

### Corollary - emulation under homomorphism

If $m\in \left(S,R\right)\to \left(\hat{S},\hat{R}\right)$ is a CRN homomorphism and a CRN stoichiomorphism, then for *any* change of rates $\hat{\iota }\in \left(\hat{S},\hat{R}\right)\to \left(\hat{S},{\hat{R}}^{\prime }\right)$, there is *some* change of rates *ι* ∈ (*S*, *R*) → (*S*, *R*′) such that $\hat{\iota }\circ m\circ \iota^{-1}\in \left(S,R^{\prime }\right)\to \left(\hat{S},{\hat{R}}^{\prime }\right)$ is a CRN homomorphism and a CRN emulation.

Proof

By the Change of Rates Theorem for any $\hat{\iota }$ there is a *ι* such that $\hat{\iota }\circ m\circ \iota^{-1}$ is a stoichiomorphism (and such that $k^{\prime }=k\cdot \frac{\hat{k^{\prime }}}{\hat{k}}$). Moreover, if *m* is a homomorphism and $\hat{\iota }$, *ι* are changes of rates, we have: $\left(\hat{\iota }\circ m\circ \iota^{-1}\right)\left(\rho \to^{k^{\prime }}\pi \right)=\left(\hat{\iota }\circ m\right)\left(\rho \to^{k}\pi \right)=\hat{\iota }\left(m\left(\rho \right)\to^{\hat{k}}m\left(\pi \right)\right)=m\left(\rho \right)\to^{\hat{k^{\prime }}}m\left(\pi \right)$, where $m\left(\rho \right)=\left(id_{\hat{S}}\circ m\circ id_{S}\right)\left(\rho \right)=\left(i\circ m\circ \iota^{-1}\right)\left(\rho \right)$ and *m*(*π*) = (*i* ∘ *m* ∘ *ι*^- 1^)(*π*) because $\hat{\iota }$, *ι* are identities on species. Moreover $k^{\prime }=k\cdot \frac{\hat{k^{\prime }}}{\hat{k}}$ where $k=\hat{k}$ by homomorphism and hence $k^{\prime }=\hat{k^{\prime }}$. Therefore $\hat{\iota }\circ m\circ \iota^{-1}$ is a homomorphism, and hence a reactant morphism. Therefore, by the Emulation Theorem, $\hat{\iota }\circ m\circ \iota^{-1}$ is an emulation.

■

This homomorphism corollary has some interesting implications in terms of finding emulation morphisms between networks. It says that if *m* is a homomorphism and we change rates in $\left(\hat{S},\;\hat{R}\right)$ then we can just *copy* the rate changes in (*S, R*) (since $\hat{\iota }\circ m\circ \iota^{-1}$ is a homomorphism) and preserve emulation. In particular, if we establish that a homomorphism/stoichiomorphism exists between networks that have all *unit rates*, then we can extend the emulation to any rate assignment, so we should first try homomorphisms with unit rates. Moreover, to find a stoichiomorphism between networks that have all unit rates, it is sufficient to check the simpler *net stoichiomorphism condition* (with the rate-free *η* instead of *φ*):

$$\forall s\in S\forall \hat{r}\in \hat{R}\;\sum_{r\in m^{-1}\left(\hat{r}\right)}\eta \left(s,r\right)=\eta \left(m\left(s\right),\hat{r}\right)$$

In fact, as exemplified in Additional file 3, all the emulation morphism in Figure 3 (which are all homomorphism) can be checked with this strategy, by checking the net stoichiomorphism condition between unit rate networks.

In summary, a stoichiomorphism $m\in \left(S,\;R\right)\to \left(\hat{S},\;\hat{R}\right)$ that is also a reactant morphism, determines an emulation for any choice of rates of $\left(\hat{S},\;\hat{R}\right)$. Those emulations can match any initial conditions of any choice of rates of $\left(\hat{S},\;\hat{R}\right)$ with some initial conditions of some choice of rates of (*S, R*).

## Abbreviations

AM: Approximate majority (algorithm); CRN: Chemical reaction network.

## Competing interests

The author declares that he has no competing interests.

## Supplementary Material

Additional file 1 The triplet model of influence. Click here for file

Additional file 2 Examples of network morphisms. Click here for file

Additional file 3 Checking some networks morphisms. Click here for file

Additional file 4 Steady states. Click here for file

Additional file 5 Lemmas and proofs. Click here for file
