# Supplementary material for: Morphisms of reaction networks that couple structure to function
Source: BMC Syst Biol. 2014 Aug 15;8:84. doi: 10.1186/1752-0509-8-84 (PMC4236760; doi:10.1186/1752-0509-8-84)
Supplement: Additional file 1 — The triplet model of influence. [file 1752-0509-8-84-S1.pdf]

Additional File 1: The triplet model of influence

An *influence network* is a graph of *influence nodes (species)* and *influence edges (reactions)*. Each influence node x is modeled as three chemical species, denoted x_0, x_1, x_2 (Figure 2). Each influence node can have four terminals: high output (solid line), low output (dashed line), activation input (ball) and inhibition input (bar). Influence edges connect two such terminals in one of the four patterns: low-to-activate, low-to-inhibit, high-to-activate, and high-to-inhibit, with at most one edge of each kind for each pair of (possibly coincident) nodes.

Each influence node x corresponds to a motif of three chemical species and four chemical reactions (Figure 2). That is, if i is a chemical species connected to the inhibition terminal, a is one connected to the activation terminal, and k_{01}, k_{12}, k_{21}, k_{10} are rates associated with the node x, we have the four reactions:

$$x_0 + i \to^{k_{01}} i + x_1, \quad x_1 + i \to^{k_{12}} i + x_2, \quad x_2 + a \to^{k_{21}} a + x_1, \quad x_1 + a \to^{k_{10}} a + x_0$$

This expansion of influence nodes into reaction motifs is sufficient to extract a chemical reaction network from any influence network, taking into account all the influence edges in the network (see Additional File 3 for examples).

We can solve the mass action equations of those four reactions at steady state, with $tot = x_0 + x_1 + x_2$, obtaining x_0 as a function of a and i:

$$x_0 = \frac{k_{10}k_{21}tot\, a^2}{k_{10}k_{21}a^2 + k_{01}k_{21}ai + k_{01}k_{12}i^2}$$

Assuming $i = tot - a$ (inhibition decreases as activation increases), we obtain x_0 as a function of a:

$$x_0 = \frac{k_{10}k_{21}tot\, a^2}{(k_{10}k_{21} - k_{01}k_{21} + k_{01}k_{12})a^2 + (k_{01}k_{21} - 2k_{01}k_{12})tot\, a + k_{01}k_{12}tot^2} = \frac{k_1 a^2}{k_2 a^2 + k_3 a + k_4}$$

This is a generalized Hill function of coefficient 2, where the coefficients k_i depend on the four reaction rates and on tot. By regulating the rates of flow through x_1 within two orders of magnitude we can obtain a range of linear, hyperbolic and sigmoid responses in the range $[0..tot]$ to linear activation $a \in [0..tot]$: note that the response range is equal to the stimulus range. Therefore, this motif is sufficiently flexible for the purpose of characterizing intended influence networks.

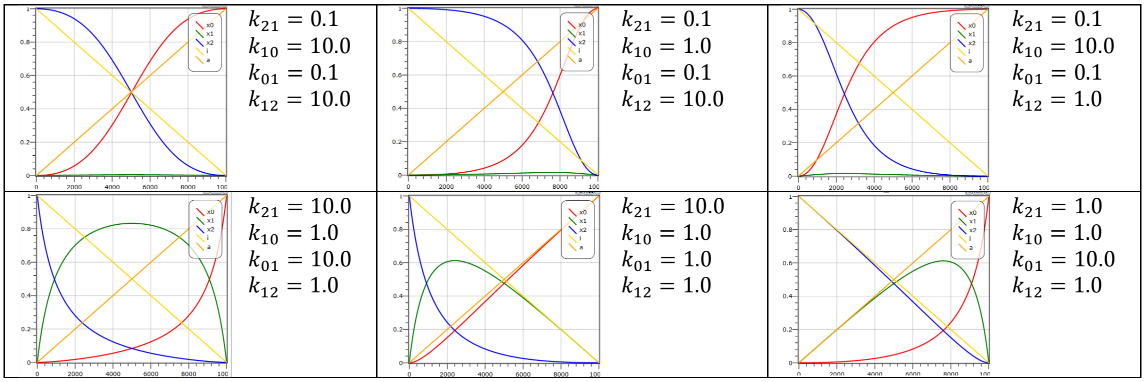

Figure A2.1. Steady state transitions from inhibited to activated with $tot = 1$ and $a \in [0..tot]$.
