# Supplementary material for: Morphisms of reaction networks that couple structure to function
Source: BMC Syst Biol. 2014 Aug 15;8:84. doi: 10.1186/1752-0509-8-84 (PMC4236760; doi:10.1186/1752-0509-8-84)
Supplement: Additional file 2 — Examples of network morphisms. [file 1752-0509-8-84-S2.pdf]

Additional File 2: Examples of network morphisms

See text around Figure 6 for an explanation of the graphical notation. Solid arrows (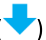) indicate emulation.

Ex.1: A simple stoichiomorphism, that is, species in the source reactions are distinct:

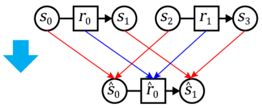

$(S,R) = (\{s_0,s_1,s_2,s_3\},\{r_0 = s_0 \to^k s_1,\ \ r_1 = s_2 \to^k s_3\})$
$(\hat{S},\hat{R}) = (\{\hat{s}_0,\hat{s}_1\},\{\hat{r}_0 = \hat{s}_0 \to^k \hat{s}_1\})$
$m(s_0) = m(s_2) = \hat{s}_0;\ \ m(s_1) = m(s_3) = \hat{s}_1;\ \ m(r_0) = m(r_1) = \hat{r}_0$

Ex.2: A homomorphism that is not a stoichiomorphism:

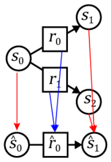

$(S,R) = (\{s_0,s_1,s_2\},\{r_0 = s_0 \to^k s_1,\ \ r_1 = s_0 \to^k s_2\})$
$(\hat{S},\hat{R}) = (\{\hat{s}_0,\hat{s}_1\},\{\hat{r}_0 = \hat{s}_0 \to^k \hat{s}_1\})$
$m(s_0) = \hat{s}_0;\ \ m(s_1) = m(s_2) = \hat{s}_1;\ \ \ m(r_0) = m(r_1) = \hat{r}_0$

Because for $s_0, \hat{r}_0$: $\Sigma_{r\in m^{-1}(\hat{r}_0)}\ \varphi(s_0,r) = -2k \neq -1k = \varphi(m(s_0),\hat{r}_0)$.

A stoichiomorphism that is not a homomorphism or a reactant morphism:

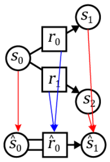

$(S,R) = (\{s_0,s_1,s_2\},\{r_0 = s_0 \to^k s_1,\ \ r_1 = s_0 \to^k s_2\})$
$(\hat{S},\hat{R}) = (\{\hat{s}_0,\hat{s}_1\},\{\hat{r}_0 = \hat{s}_0 + \hat{s}_0 \to^k \hat{s}_1\})$
$m(s_0) = \hat{s}_0;\ \ m(s_1) = m(s_2) = \hat{s}_1;\ \ \ m(r_0) = m(r_1) = \hat{r}_0$

Another homomorphism that is not a stoichiomorphism:

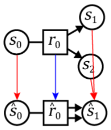

$(S,R) = (\{s_0,s_1,s_2\},\{r_0 = s_0 \to^k s_1 + s_2\})$
$(\hat{S},\hat{R}) = (\{\hat{s}_0,\hat{s}_1\},\{\hat{r}_0 = \hat{s}_0 \to^k \hat{s}_1 + \hat{s}_1\})$
$m(s_0) = \hat{s}_0;\ \ m(s_1) = m(s_2) = \hat{s}_1;\ \ \ m(r_0) = \hat{r}_0$

Because for $s_1, \hat{r}_0$: $\Sigma_{r\in m^{-1}(\hat{r}_0)}\ \varphi(s_1,r) = k \neq 2k = \varphi(m(s_0),\hat{r}_0)$.

A stoichiomorphism that is not a homomorphisms, but is a reactant morphism:

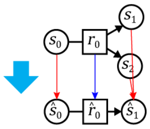

$(S,R) = (\{s_0,s_1,s_2\},\{r_0 = s_0 \to^k s_1 + s_2\})$
$(\hat{S},\hat{R}) = (\{\hat{s}_0,\hat{s}_1\},\{\hat{r}_0 = \hat{s}_0 \to^k \hat{s}_1\})$
$m(s_0) = \hat{s}_0;\ \ m(s_1) = m(s_2) = \hat{s}_1;\ \ \ m(r_0) = \hat{r}_0$

Another stoichiomorphism that is not a homomorphisms but is a reactant morphism:

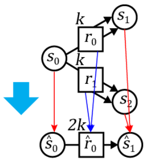

$(S,R) = (\{s_0,s_1,s_2\},\{r_0 = s_0 \to^k s_1 + s_1,\ \ r_1 = s_0 \to^k s_2 + s_2\})$
$(\hat{S},\hat{R}) = (\{\hat{s}_0,\hat{s}_1\},\{\hat{r}_0 = \hat{s}_0 \to^{2k} \hat{s}_1\})$
$m(s_0) = \hat{s}_0;\ \ m(s_1) = m(s_2) = \hat{s}_1;\ \ \ m(r_0) = m(r_1) = \hat{r}_0$

Ex.3: A stoichiomorphism that is not surjective on species or reactions, and not completely trivial because $\hat{s}_0$ occurs in $\hat{r}_1$, so an 'extra' reaction uses a 'non-extra' species:

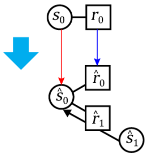

$(S,R) = (\{s_0\},\{r_0 = s_0 \to^k\})$
$(\hat{S},\hat{R}) = (\{\hat{s}_0,\hat{s}_1\},\{\hat{r}_0 = \hat{s}_0 \to^k,\ \ \hat{r}_1 = \hat{s}_0 + \hat{s}_1 \to^k \hat{s}_0\})$
$m(s_0) = \hat{s}_0;\ \ \ \ m(r_0) = \hat{r}_0$
$m^{-1}(\hat{r}_1) = \emptyset,\ \ \Sigma_{r\in m^{-1}(\hat{r}_1)}\ \varphi(s_0,r) = 0 = \varphi(\hat{s}_0,\hat{r}_1)$

Ex.4: A homomorphism and stoichiomorphism that is not injective.

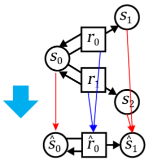

$(S,R) = (\{s_0,s_1,s_2\},\{r_0 = s_0 \to^k s_0 + s_1,\ \ r_1 = s_0 \to^k s_0 + s_2\})$
$(\hat{S},\hat{R}) = (\{\hat{s}_0,\hat{s}_1\},\{\hat{r}_0 = \hat{s}_0 \to^k \hat{s}_0 + \hat{s}_1\})$
$m(s_0) = \hat{s}_0;\ \ m(s_1) = m(s_2) = \hat{s}_1;\ \ \ \ m(r_0) = m(r_1) = \hat{r}_0$

Ex.5: A homomorphism and stoichiomorphism that is not injective on species in the same reaction. If we remove r_1 it is still a homomorphism but no longer a stoichiomorphism.

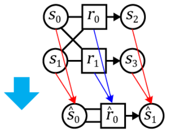

$$(S,R) = (\{s_0,s_1,s_2,s_3\},\{r_0 = s_0 + s_1 \to^k s_2,\ \ r_1 = s_0 + s_1 \to^k s_3\})$$
$$(\hat{S},\hat{R}) = (\{\hat{s}_0,\hat{s}_1\},\{\hat{r}_0 = 2\cdot\hat{s}_0 \to^k \hat{s}_1\})$$
$$m(s_0) = m(s_1) = \hat{s}_0;\ \ m(s_2) = m(s_3) = \hat{s}_1;\ \ m(r_0) = m(r_1) = \hat{r}_0$$
$$\textstyle\sum_{r\in m^{-1}(\hat{r}_0)} \varphi(s_0,r) = -2k = \varphi(\hat{s}_0,\hat{r}_0)$$

Ex.6: Here m fails to be a stoichiomorphism, when attempting to map a non-loop onto a loop of reactions.

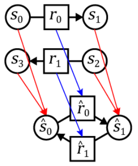

$$(S,R) = (\{s_0,s_1,s_2,s_3\},\{r_0 = s_0 \to^k s_1,\ \ r_1 = s_3 \to^k s_2\})$$
$$(\hat{S},\hat{R}) = (\{\hat{s}_0,\hat{s}_1\},\{\hat{r}_0 = \hat{s}_0 \to^k \hat{s}_1,\ \ \hat{r}_1 = \hat{s}_1 \to^k \hat{s}_0\})$$
$$m(s_0) = m(s_2) = \hat{s}_0;\ \ m(s_1) = m(s_3) = \hat{s}_1;\ \ \ m(r_0) = \hat{r}_0;\ \ m(r_1) = \hat{r}_1$$

Because for $s_0, \hat{r}_1$: $\sum_{r\in m^{-1}(\hat{r}_1)} \varphi(s_0,r) = \varphi(s_0,r_1) = 0 \neq 1k = \varphi(\hat{s}_0,\hat{r}_1) = \varphi(m(s_0),\hat{r}_1)$.

Ex.7: Similarly m fails to be a stoichiomorphism when mapping a catalysis to an autocatalysis.

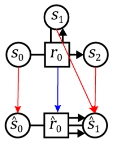

$$(S,R) = (\{s_0,s_1,s_2\},\{r_0 = s_0 + s_1 \to^k s_1 + s_2\})$$
$$(\hat{S},\hat{R}) = (\{\hat{s}_0,\hat{s}_1\},\{\hat{r}_0 = \hat{s}_0 + \hat{s}_1 \to^k \hat{s}_1 + \hat{s}_1\})$$
$$m(s_0) = \hat{s}_0;\ \ m(s_1) = m(s_2) = \hat{s}_1;\ \ \ m(r_0) = \hat{r}_0$$

Because for $s_1, \hat{r}_0$: $\sum_{r\in m^{-1}(\hat{r}_0)} \varphi(s_1,r) = \varphi(s_1,r_0) = 0 \neq 1k = \varphi(\hat{s}_1,\hat{r}_0) = \varphi(m(s_1),\hat{r}_0)$.

Ex.8: But m is a stoichiomorphism when mapping mutual catalysis to autocatalysis:

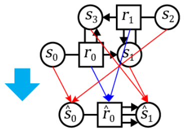

$$(S,R) = (\{s_0,s_1,s_2,s_3\},\{r_0 = s_0 + s_3 \to^k s_3 + s_1,\ r_1 = s_2 + s_1 \to^k s_1 + s_3\})$$
$$(\hat{S},\hat{R}) = (\{\hat{s}_0,\hat{s}_1\},\{\hat{r}_0 = \hat{s}_0 + \hat{s}_1 \to^k \hat{s}_1 + \hat{s}_1\})$$
$$m(s_0) = m(s_2) = \hat{s}_0;\ \ m(s_1) = m(s_3) = \hat{s}_1;\ \ \ m(r_0) = m(r_1) = \hat{r}_0$$

For similar examples, consider cycles of unimolecular reactions: a cycle of length 2 can be mapped by a stoichiomorphism to a cycle of length 1, and a cycle of length 4 (but not of length 3) can be mapped on a cycle of length 2.

Ex.9: An important way in which a homomorphism or reactant morphism may fail to be a stoichiomorphism is due to 'reaction chaining' under the morphism. Below is a simple case, but this may easily happen for example when collapsing a 2-loop into a 1-loop as in Ex.8, but where there is also a reaction connected to just one of the loop species that gets chained to the common species under the loop collapse.

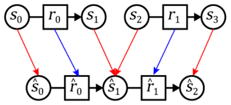

$$(S,R) = (\{s_0,s_1,s_2,s_3\},\{r_0 = s_0 \to^k s_1,\ r_1 = s_2 \to^k s_3\})$$
$$(\hat{S},\hat{R}) = (\{\hat{s}_0,\hat{s}_1,\hat{s}_2\},\{\hat{r}_0 = \hat{s}_0 \to^k \hat{s}_1,\ \hat{r}_1 = \hat{s}_1 \to^k \hat{s}_2\})$$
$$m(s_0) = \hat{s}_0;\ \ m(s_1) = m(s_2) = \hat{s}_1;\ \ m(s_3) = \hat{s}_2;\ \ m(r_0) = \hat{r}_0;\ \ m(r_1) = \hat{r}_1$$

Then for $s_2, \hat{r}_0$: $\sum_{r\in m^{-1}(\hat{r}_0)} \varphi(s_2,r) = \varphi(s_2,r_0) = 0 \neq 1k = \varphi(\hat{s}_1,\hat{r}_0) = \varphi(m(s_2),\hat{r}_0)$. An even simpler, degenerate case, is as above but where there are no $s_3, r_1, \hat{s}_2, \hat{r}_1$, but still $m(s_2) = \hat{s}_1$.

Ex.10: A stoichiomorphism that is a reactant morphism but not a homomorphism (rates vary). It yields an emulation since concentrations of s_0 and s_1 decrease like $\hat{s}_0$ from equal initial conditions.

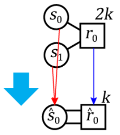

$$(S,R) = (\{s_0,s_1\},\{r_0 = s_0 + s_1 \to^{2k}\})$$
$$(\hat{S},\hat{R}) = (\{\hat{s}_0\},\{\hat{r}_0 = \hat{s}_0 + \hat{s}_0 \to^k\})$$
$$m(s_0) = m(s_1) = \hat{s}_0;\ \ m(r_0) = \hat{r}_0$$

$$\textstyle\sum_{r\in m^{-1}(\hat{r}_0)} \varphi(s_0,r) = -2k = \varphi(m(s_0),\hat{r}_0)$$
$$\textstyle\sum_{r\in m^{-1}(\hat{r}_0)} \varphi(s_1,r) = -2k = \varphi(m(s_1),\hat{r}_0)$$

This is an example where unit rates are not sufficient. To change rates while maintaining an emulation, choose a new rate k' for $\hat{r}_0$. Then, according to the construction in the Change of Rates Theorem, we can choose a rate $(2k)\cdot\frac{k'}{k} = 2k'$ for r_0, for which we still have a stoichiomorphism and an emulation over the modified networks.

Ex.11: Another stoichiomorphism that is a reactant morphism but not a homomorphism (rates vary). It yields an emulation since concentrations of s_1, s_2 and $\hat{s}_1$ do not change and s_0 can decrease like $\hat{s}_0$.

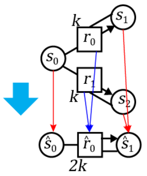

$$(S,R) = (\{s_0,s_1,s_2\},\{r_0 = s_0 + s_1 \to^k s_1,\ r_1 = s_0 + s_2 \to^k s_2\})$$
$$(\hat{S},\hat{R}) = (\{\hat{s}_0,\hat{s}_1\},\{\hat{r}_0 = \hat{s}_0 + \hat{s}_1 \to^{2k} \hat{s}_1\})$$
$$m(s_0) = \hat{s}_0;\ \ m(s_1) = m(s_2) = \hat{s}_1;\quad m(r_0) = m(r_1) = \hat{r}_0$$

$$\textstyle\sum_{r\in m^{-1}(\hat{r}_0)} \varphi(s_0,r) = -2k = \varphi(m(s_0),\hat{r}_0)$$
$$\textstyle\sum_{r\in m^{-1}(\hat{r}_0)} \varphi(s_1,r) = 0 = \varphi(m(s_1),\hat{r}_0)$$
$$\textstyle\sum_{r\in m^{-1}(\hat{r}_0)} \varphi(s_2,r) = 0 = \varphi(m(s_2),\hat{r}_0)$$

Ex.12: Examples 1, 2.4, 2.5, 3, 4, 5, 8 are further morphisms that yield emulations.

Ex.13: Counterexamples to the inverse of the Emulation Theorem. This statement is not true: A morphism that is a reactant morphism and an emulation is a stoichiomorphism. The counterexample is based on two distinct reactions with the same reagent; in fact, the statement above holds if the target CNR has no two reactions with the same reagents (see Additional File 5, Only-If Propositions).

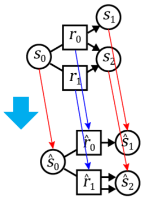

$$(S,R) = (\{s_0,s_1,s_2\},\{r_0 = s_0 \to^k s_1 + s_2, r_1 = s_0 \to^k s_2\})$$
$$(\hat{S},\hat{R}) = (\{\hat{s}_0,\hat{s}_1,\hat{s}_2\},\{\hat{r}_0 = \hat{s}_0 \to^k \hat{s}_1,\ \hat{r}_1 = \hat{s}_0 \to^k \hat{s}_2 + \hat{s}_2\})$$
$$m(s_0) = \hat{s}_0;\ \ m(s_1) = \hat{s}_1;\ \ m(s_2) = \hat{s}_2;$$
$$m(r_0) = \hat{r}_0\ ;\ m(r_1) = \hat{r}_1$$

This is a reactant morphism and emulation but not stoichiomorphism for $s_2,\hat{r}_1$ and $s_2,\hat{r}_2$. Moreover, requiring the reactant morphism to be a homomorphism does not help, as the following counterexample shows:

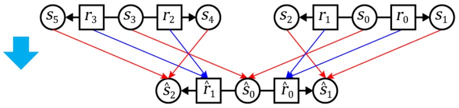

$$(S,R) = (\{s_0,s_1,s_2,s_3,s_4,s_5\},\{r_0 = s_0 \to^k s_1, r_1 = s_0 \to^k s_2,\ r_2 = s_3 \to^k s_4,\ r_3 = s_3 \to^k s_5\})$$
$$(\hat{S},\hat{R}) = (\{\hat{s}_0,\hat{s}_1,\hat{s}_2\},\{\hat{r}_0 = \hat{s}_0 \to^k \hat{s}_1,\ \hat{r}_1 = \hat{s}_0 \to^k \hat{s}_2\})$$
$$m(s_0) = m(s_3) = \hat{s}_0;\ \ m(s_1) = m(s_2) = \hat{s}_1;\ \ m(s_4) = m(s_5) = \hat{s}_2;$$
$$m(r_0) = m(r_1) = \hat{r}_0\ ;\ m(r_2) = m(r_3) = \hat{r}_1$$

This is a homomorphism and an emulation (e.g., each of $s_0, s_3, \hat{s}_0$ decrease at rate $-2k$), but not a stoichiomorphism because for $s_0,\hat{r}_0$: $\sum_{r\in m^{-1}(\hat{r}_0)} \varphi(s_0,r) = -2k \neq -k = \varphi(m(s_0),\hat{r}_0)$.

Moreover, consider the morphism over one species and one reaction such that $m(s) = \hat{s}$ and $m(s \to^k s) = 2\hat{s} \to^k 2\hat{s}$. This is an emulation and a stoichiomorphism, but not a reactant morphism.

Ex.14: Another stoichiomorphism that is not a homomorphisms, but is a reactant morphism:

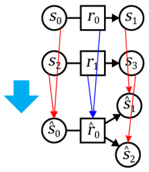

$$(S,R) = (\{s_0,s_1,s_2,s_3\},\{r_0 = s_0 \to^k s_1, r_1 = s_2 \to^k s_3\})$$
$$(\hat{S},\hat{R}) = (\{\hat{s}_0,\hat{s}_1,\hat{s}_2\},\{\hat{r}_0 = \hat{s}_0 \to^k \hat{s}_1 + \hat{s}_2\})$$
$$m(s_0) = m(s_2) = \hat{s}_0;\ \ m(s_1) = \hat{s}_1;\ \ m(s_3) = \hat{s}_2;$$
$$m(r_0) = m(r_1) = \hat{r}_0$$

In general, there is a stoichiomorphism and reactant morphism between a tree and its set of paths.
