# Supplementary material for: Morphisms of reaction networks that couple structure to function
Source: BMC Syst Biol. 2014 Aug 15;8:84. doi: 10.1186/1752-0509-8-84 (PMC4236760; doi:10.1186/1752-0509-8-84)
Supplement: Additional file 3 — Checking some networks morphisms. [file 1752-0509-8-84-S3.pdf]

Additional File 3: Checking some networks morphisms

Stoichiomorphism & homomorphism from MI to AM

This stoichiomorphism maps $z, \sim y \rightarrow x$.

	Species	Reactions	Reaction Network	Influence Network
AM	x_0, x_1, x_2	$am_0 = x_2 + x_0 \rightarrow x_0 + x_1$<br>$am_1 = x_1 + x_0 \rightarrow x_0 + x_0$<br>$am_2 = x_0 + x_2 \rightarrow x_2 + x_1$<br>$am_3 = x_1 + x_2 \rightarrow x_2 + x_2$	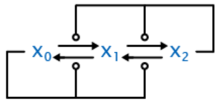	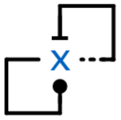
MI	$y_0, y_1, y_2,$<br>z_0, z_1, z_2	$mi_0 = y_0 + z_0 \rightarrow z_0 + y_1$<br>$mi_1 = y_1 + z_0 \rightarrow z_0 + y_2$<br>$mi_2 = y_2 + y_0 \rightarrow y_0 + y_1$<br>$mi_3 = y_1 + y_0 \rightarrow y_0 + y_0$<br>$mi_4 = z_2 + z_0 \rightarrow z_0 + z_1$<br>$mi_5 = z_1 + z_0 \rightarrow z_0 + z_0$<br>$mi_6 = z_0 + y_0 \rightarrow y_0 + z_1$<br>$mi_7 = z_1 + y_0 \rightarrow y_0 + z_2$	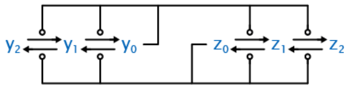	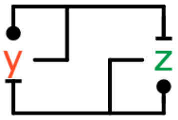

The species map $m(s)$ below (outer columns) yields a homomorphism over reactions. We then check that m preserves net stoichiometry $\eta(s,r)$, for all species in MI and all reactions in AM. For example the top left cell checks that $\eta(z_0, mi_0) + \eta(z_0, mi_4) = 0 = \eta(x_0, am_0)$.

		$m^{-1}(\hat{r}) \subseteq \text{MI}$					
		mi_0, mi_4	mi_1, mi_5	mi_2, mi_6	mi_3, mi_7		
$\forall s \in \text{MI}$	z_0	0	1	-1	0	x_0	$m(s) \in \text{AM}$
	z_1	1	-1	1	-1	x_1	
	z_2	-1	0	0	1	x_2	
	y_0	-1	0	0	1	x_2	
	y_1	1	-1	1	-1	x_1	
	y_2	0	1	-1	0	x_0	
		am_0	am_1	am_2	am_3		
		$\forall \hat{r} \in \text{AM}$					

Therefore, by the stoichiomorphism theorem, trajectories of MI that start with $\forall s \in \{z_0, z_1, z_2, y_2, y_1, y_0\}$ $[m(s)] = [s]$, coincide with trajectories of AM.

AM with unit rates and initial state: $x_0 = 2,\ x_1 = 0,\ x_2 = 1$	MI with unit rates and initial state: $y_2 = z_0 = 2,\ y_1 = z_1 = 0,\ y_0 = z_2 = 1$
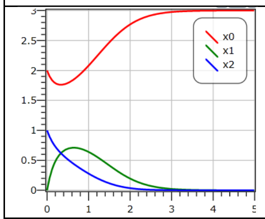	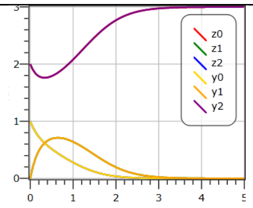

Stoichiomorphism & homomorphism from QI to MI

This stoichiomorphism maps $z, r \to z$ and $y, s \to y$.

	Species	Reactions		Influence Network
MI	$y_0, y_1, y_2,$ z_0, z_1, z_2	$mi_0 = y_0 + z_0 \to z_0 + y_1$<br>$mi_1 = y_1 + z_0 \to z_0 + y_2$<br>$mi_2 = y_2 + y_0 \to y_0 + y_1$<br>$mi_3 = y_1 + y_0 \to y_0 + y_0$<br>$mi_4 = z_2 + z_0 \to z_0 + z_1$<br>$mi_5 = z_1 + z_0 \to z_0 + z_0$<br>$mi_6 = z_0 + y_0 \to y_0 + z_1$<br>$mi_7 = z_1 + y_0 \to y_0 + z_2$		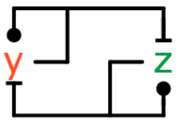
QI	$y_0, y_1, y_2,$ $z_0, z_1, z_2,$ $s_0, s_1, s_2,$ r_0, r_1, r_2	$qi_0 = s_0 + z_0 \to z_0 + s_1$<br>$qi_1 = s_1 + z_0 \to z_0 + s_2$<br>$qi_2 = s_2 + y_0 \to y_0 + s_1$<br>$qi_3 = s_1 + y_0 \to y_0 + s_0$<br>$qi_4 = r_2 + z_0 \to z_0 + r_1$<br>$qi_5 = r_1 + z_0 \to z_0 + r_0$<br>$qi_6 = r_0 + y_0 \to y_0 + r_1$<br>$qi_7 = r_1 + y_0 \to y_0 + r_2$	$qi_8 = y_0 + r_0 \to r_0 + y_1$<br>$qi_9 = y_1 + r_0 \to r_0 + y_2$<br>$qi_{10} = y_2 + s_0 \to s_0 + y_1$<br>$qi_{11} = y_1 + s_0 \to s_0 + y_0$<br>$qi_{12} = z_2 + r_0 \to r_0 + z_1$<br>$qi_{13} = z_1 + r_0 \to r_0 + z_0$<br>$qi_{14} = z_0 + s_0 \to s_0 + z_1$<br>$qi_{15} = z_1 + s_0 \to s_0 + z_2$	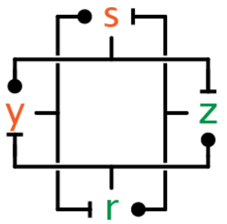

The species map $m(s)$ below (outer columns) yields a homomorphism over reactions. We then check that m preserves net stoichiometry $\eta(s, r)$, for all species in MI and all reactions in AM. For example the top left cell checks that $\eta(z_0, qi_0) + \eta(z_0, qi_8) = 0 = \eta(z_0, mi_0)$.

		$m^{-1}(\hat{r})$									
		qi_0, qi_8	qi_1, qi_9	qi_2, qi_{10}	qi_3, qi_{11}	qi_4, qi_{12}	qi_5, qi_{13}	qi_6, qi_{14}	qi_7, qi_{15}		
$\forall s \in$ QI	z_0	0	0	0	0	0	1	-1	0	z_0	$m(s)$
	z_1	0	0	0	0	1	-1	1	-1	z_1	
	z_2	0	0	0	0	-1	0	0	1	z_2	
	r_0	0	0	0	0	0	1	-1	0	z_0	
	r_1	0	0	0	0	1	-1	1	-1	z_1	
	r_2	0	0	0	0	-1	0	0	1	z_2	
	y_0	-1	0	0	1	0	0	0	0	y_0	
	y_1	1	-1	1	-1	0	0	0	0	y_1	
	y_2	0	1	-1	0	0	0	0	0	y_2	
	s_0	-1	0	0	1	0	0	0	0	y_0	
	s_1	1	-1	1	-1	0	0	0	0	y_1	
	s_2	0	1	-1	0	0	0	0	0	y_2	
		mi_0	mi_1	mi_2	mi_3	mi_4	mi_5	mi_6	mi_7		
		$\forall \hat{r} \in$ MI									

We give an example of how this stoichiomorphism can yield an emulation from QI to MI for heterogeneous rates and initial conditions.

MI with heterogeneous rates and initial conditions	QI with MI-copied rates and initial conditions
$z_0 = 2, z_1 = 0, z_2 = 1, y_0 = 0.9, y_1 = 0.2, y_2 = 1.9$<br>$mi_0 = 0.7, mi_1 = 0.8, mi_2 = 0.9, mi_3 = 1.0, mi_4 = 0.5, mi_5 = 0.6, mi_6 = 1.1, mi_7 = 1.2$	$z_0 = 2, z_1 = 0, z_2 = 1, y_0 = 0.9, y_1 = 0.2, y_2 = 1.9$<br>$r_0 = 2, r_1 = 0, r_2 = 1, s_0 = 0.9, s_1 = 0.2, s_2 = 1.9$<br>$qi_0 = 0.7, qi_1 = 0.8, qi_2 = 0.9, qi_3 = 1.0, qi_4 = 0.5, qi_5 = 0.6, qi_6 = 1.1, qi_7 = 1.2$<br>$qi_8 = 0.7, qi_9 = 0.8, qi_{10} = 0.9, qi_{11} = 1.0, qi_{12} = 0.5, qi_{13} = 0.6, qi_{14} = 1.1, qi_{15} = 1.2$
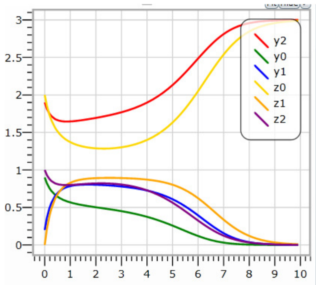	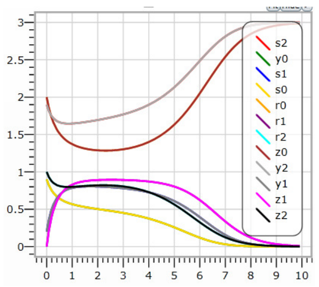
