# Supplementary material for: Morphisms of reaction networks that couple structure to function
Source: BMC Syst Biol. 2014 Aug 15;8:84. doi: 10.1186/1752-0509-8-84 (PMC4236760; doi:10.1186/1752-0509-8-84)
Supplement: Additional file 4 — Steady states. [file 1752-0509-8-84-S4.pdf]

Additional File 4: Steady states

It can be shown that AM and SI have each 4 steady states, and MI has an additional class of unstable steady states (bottom row). Here all rates are 1.0 and K is the total amount of each triplet of species:

Steady states	AM	SI	MI
unstable	$x_0 = x_1 = x_2 = K/3$	$z_0 = z_1 = z_2 = y_0 = y_1 = y_2 = K/3$	$z_0 = z_1 = z_2 = y_0 = y_1 = y_2 = K/3$
stable	$x_0 = K,\ \ x_2 = x_1 = 0$	$z_0 = y_2 = K,\ \ z_1 = z_2 = y_0 = y_1 = 0$	$z_0 = y_2 = K,\ \ z_1 = z_2 = y_0 = y_1 = 0$
stable	$x_2 = K,\ \ x_0 = x_1 = 0$	$z_2 = y_0 = K,\ \ z_0 = z_1 = y_1 = y_2 = 0$	$z_2 = y_0 = K,\ \ z_0 = z_1 = y_1 = y_2 = 0$
unstable	$x_1 = K,\ \ x_2 = x_0 = 0$	$z_1 = y_1 = K,\ \ z_0 = z_2 = y_0 = y_2 = 0$	$z_1 + z_2 = y_1 + y_2 = K,\ \ z_0 = y_0 = 0$
	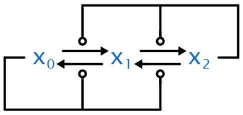	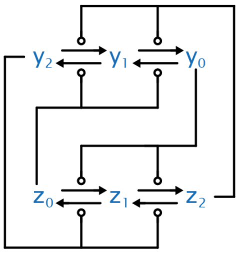	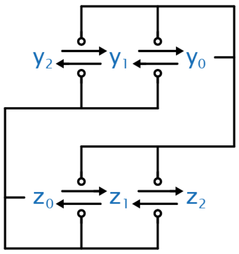

We can see that the steady states of AM are preserved under the species mappings shown in Figure 3 above. More generally, by the Steady State Proposition, we can infer that the bistability of AM is preserved by all the networks in Figure 3 that are connected to AM by blue arrows: for each, two specific distinct steady states can be derived from the mappings, without need to compute their steady states separately.
