# Supplementary material for: Morphisms of reaction networks that couple structure to function
Source: BMC Syst Biol. 2014 Aug 15;8:84. doi: 10.1186/1752-0509-8-84 (PMC4236760; doi:10.1186/1752-0509-8-84)
Supplement: Additional file 5 — Lemmas and proofs. [file 1752-0509-8-84-S5.pdf]

# Additional File 5: Lemmas and proofs

## Morphism lemmas

### *Definition - Reactant morphism*

$m \in (S,R) \to (\hat{S},\hat{R})$ is a CRN reactant morphism iff

$$\forall \rho \to^k \pi \in R \quad \exists \hat{\pi},\hat{k} \quad m_{\mathcal{R}}(\rho \to^k \pi) = m_{\mathcal{S}}(\rho) \to^{\hat{k}} \hat{\pi}$$

### *Lemma - Reactant morphism*

$m \in (S,R) \to (\hat{S},\hat{R})$ is a CRN reactant morphism iff $\forall r \in R \;\; m(1^{st}(r)) = 1^{st}(m(r))$.

*Proof*

Let $r = \rho \to^k \pi$ and $m(\rho \to^k \pi) = m(\rho) \to^{\hat{k}} \hat{\pi}$ for some $\hat{\pi},\hat{k}$. Then $m(1^{st}(\rho \to^k \pi)) = m(\rho) = 1^{st}(m(\rho) \to^{\hat{k}} \hat{\pi}) = 1^{st}(m(\rho \to^k \pi))$. Conversely, Let $r = \rho \to^k \pi$ and $m(r) = \hat{\rho} \to^{\hat{k}} \hat{\pi}$ and $m(1^{st}(r)) = 1^{st}(m(r))$. Then $m(1^{st}(r)) = m(1^{st}(\rho \to^k \pi)) = m(\rho)$ and $1^{st}\big(m(r)\big) = 1^{st}\big(\hat{\rho} \to^{\hat{k}} \hat{\pi}\big) = \hat{\rho}$. Hence $m(\rho) = \hat{\rho}$ and there exists $\hat{\pi},\hat{k}$ such that $m(\rho \to^k \pi) = \hat{\rho} \to^{\hat{k}} \hat{\pi} = m(\rho) \to^{\hat{k}} \hat{\pi}$.

∎

### *Definition - Homomorphism*

$m \in (S,R) \to (\hat{S},\hat{R})$ is a CRN homomorphism iff

$$\forall \rho \to^k \pi \in R \qquad m_{\mathcal{R}}(\rho \to^k \pi) = m_{\mathcal{S}}(\rho) \to^k m_{\mathcal{S}}(\pi)$$

### *Lemma - Homomorphism*

If $m \in (S,R) \to (\hat{S},\hat{R})$ is a CRN homomorphism then:

$$\forall \hat{s} \in \hat{S} \;\; \forall r \in R \;\; \varphi(\hat{s}, m(r)) = \textstyle\sum_{s \in m^{-1}(\hat{s})} \varphi(s,r)$$

*Proof*

Assume $m$ is a homomorphism. Take any $r = \rho \to^k \pi \in R$ and $\hat{s} \in \hat{S}$ then:

$$\begin{aligned}
&\varphi(\hat{s}, m(r)) \\
&= \varphi\big(\hat{s}, m(\rho) \to^k m(\pi)\big) \\
&= k \cdot (m(\pi)_{\hat{s}} - m(\rho)_{\hat{s}}) \\
&= k \cdot \big(\big(\textstyle\sum_{s \in m^{-1}(\hat{s})} \pi_s\big) - \big(\textstyle\sum_{s \in m^{-1}(\hat{s})} \rho_s\big)\big) \\
&= k \cdot \textstyle\sum_{s \in m^{-1}(\hat{s})} (\pi_s - \rho_s) \\
&= \textstyle\sum_{s \in m^{-1}(\hat{s})} k \cdot (\pi_s - \rho_s) \\
&= \textstyle\sum_{s \in m^{-1}(\hat{s})} \varphi(s, \rho \to^k \pi) \\
&= \textstyle\sum_{s \in m^{-1}(\hat{s})} \varphi(s, r)
\end{aligned}$$

∎

*Remark – Weakness of homomorphism lemma*

The converse of that lemma is not true. The CRN morphism $m(s) = \hat{s}$, $m(r) = m(1 \cdot s \to 1 \cdot s) = 2 \cdot \hat{s} \to 2 \cdot \hat{s}$, is such that $\varphi\big(\hat{s}, m(r)\big) = 0 = \varphi(s, r)$. But $m(r) \neq m(1 \cdot s) \to m(1 \cdot s) = 1 \cdot \hat{s} \to 1 \cdot \hat{s}$, hence $m$ is not a homomorphism nor a reactant morphism.

Therefore the (weaker) property of homomorphisms derived in this lemma is not sufficient to prove the Mass Action Lemma (requiring a reactant morphism), and therefore also not the Emulation Theorem. Rather, the Mass Action Lemma is proven from the reactant morphism definition, which is a consequence of the homomorphism definition; both imply the reactant morphism property $m(1^{st}(r)) = 1^{st}(m(r))$ which is sufficient for the theorems.

*Lemma - Homomorphism and stoichiomorphism*

If $m \in (S, R) \to \big(\hat{S}, \hat{R}\big)$ is a CRN homomorphism and stoichiomorphism then:

$$\forall s \in S \;\; \forall r \in R \;\; \textstyle\sum_{\acute{s} \in m^{-1}(m(s))} \varphi(\acute{s}, r) = \varphi(m(s), m(r)) = \textstyle\sum_{\acute{r} \in m^{-1}(m(r))} \varphi(s, \acute{r})$$

*Proof*

Assume $m$ is a homomorphism, then: $\forall \hat{s} \in \hat{S} \;\; \forall r \in R \;\; \varphi(\hat{s}, m(r)) = \sum_{s \in m^{-1}(\hat{s})} \varphi(s, r)$.

Assume $m$ is a stoichiomorphism, then: $\forall s \in S \;\; \forall \hat{r} \in \hat{R} \;\; \sum_{r \in m^{-1}(\hat{r})} \varphi(s, r) = \varphi(m(s), \hat{r})$.

Take any $s \in S$ and $r \in R$. Consider $m(s) \in \hat{S}$ and $m(r) \in \hat{R}$.

By the first assumption $\sum_{\acute{s} \in m^{-1}(m(s))} \varphi(\acute{s}, r) = \varphi(m(s), m(r))$.

By the second assumption $\sum_{\acute{r} \in m^{-1}(m(r))} \varphi(s, \acute{r}) = \varphi(m(s), m(r))$.

■

## Fiber lemma

*Lemma – Fiber lemma for big operators*

Let $(\oplus, \varepsilon)$ be a commutative monoid over a set $B$. For $h \in C \to B$ and $I \subseteq C$ a finite set, the big-operator notation $\bigoplus_{i \in I} h(i)$ is defined inductively on $I$ as follows (where $I \backslash c = I - \{c\}$):

$$\bigoplus_{i \in \emptyset} h(i) = \varepsilon$$

$$\bigoplus_{i \in I} h(i) = h(c) \oplus (\bigoplus_{i \in I \backslash c} h(i)) \quad \text{with } c \in I$$

(Since $(\oplus, \varepsilon)$ is a commutative monoid, the choice function used does not matter.)

Then, for any finite $\hat{A}$ and $A$, any $f \in A \to \hat{A}$, and any $g \in A \times \hat{A} \to B$, we have:

$$\bigoplus_{\hat{a} \in \hat{A}} \bigoplus_{a \in f^{-1}(\hat{a})} g(a, \hat{a}) = \bigoplus_{a \in A} g\big(a, f(a)\big)$$

*Proof*

By induction on the finite size of $\hat{A}$. If $\hat{A} = \emptyset$ and $f \in A \to \hat{A}$ then $A = \emptyset$, hence by definition of big-operator:

$$\bigoplus_{\hat{a} \in \emptyset} \bigoplus_{a \in f^{-1}(\hat{a})} g(a, \hat{a}) = \varepsilon = \bigoplus_{a \in \emptyset} g\big(a, f(a)\big)$$

If instead there is some $\acute{a} \in \hat{A}$, since $f^{-1}$ partitions $A$, then $f \in (A - f^{-1}(\acute{a})) \to \hat{A} \backslash \acute{a}$ (where $f^{-1}(\acute{a})$ can be empty if $f$ is not surjective, but $A$ need not decrease in size in the induction step). Moreover any $g \in A \times \hat{A} \to B$ is also a $g \in (A - f^{-1}(\acute{a})) \times \hat{A} \backslash \acute{a} \to B$. We can thus instantiate the universal quantifiers in the statement with, respectively, $\hat{A} \backslash \acute{a}$, $A - f^{-1}(\acute{a})$, $f$, and $g$, to obtain an induction hypothesis for the smaller $\hat{A} \backslash \acute{a}$:

$$\bigoplus_{\hat{a} \in \hat{A} \backslash \acute{a}} \bigoplus_{a \in f^{-1}(\hat{a})} g(a, \hat{a}) = \bigoplus_{a \in A - f^{-1}(\acute{a})} g\big(a, f(a)\big)$$

We need to show under the current assumptions, including $\acute{a} \in \hat{A}$, that:

$$\bigoplus_{\hat{a} \in \hat{A}} \bigoplus_{a \in f^{-1}(\hat{a})} g(a, \hat{a}) = \bigoplus_{a \in A} g\big(a, f(a)\big)$$

Now, if $a \in f^{-1}(\acute{a})$ then by definition $\acute{a} = f(a)$. Hence (and this holds also if $f^{-1}(\acute{a}) = \emptyset$):

$$\bigoplus_{a \in f^{-1}(\acute{a})} g(a, \acute{a}) = \bigoplus_{a \in f^{-1}(\acute{a})} g\big(a, f(a)\big)$$

Summing those two terms to the two sides of the induction hypothesis yields:

$$(\bigoplus_{a \in f^{-1}(\acute{a})} g(a, \acute{a})) \oplus (\bigoplus_{\hat{a} \in \hat{A} \backslash \acute{a}} \bigoplus_{a \in f^{-1}(\hat{a})} g(a, \hat{a}))$$
$$= (\bigoplus_{a \in f^{-1}(\acute{a})} g\big(a, f(a)\big)) \oplus (\bigoplus_{a \in A - f^{-1}(\acute{a})} g\big(a, f(a)\big))$$

The left hand side, by definition of big-operator with $\acute{a} \in \hat{A}$, yields:

$$\left(\bigoplus_{a \in f^{-1}(\acute{a})} g(a, \acute{a})\right) \oplus \left(\bigoplus_{\hat{a} \in \hat{A} \backslash \acute{a}} \bigoplus_{a \in f^{-1}(\hat{a})} g(a, \hat{a})\right) = \bigoplus_{\hat{a} \in \hat{A}} \bigoplus_{a \in f^{-1}(\hat{a})} g(a, \hat{a})$$

The right hand side yields:

$$\left(\bigoplus_{a \in f^{-1}(\acute{a})} g\big(a, f(a)\big)\right) \oplus \left(\bigoplus_{a \in A - f^{-1}(\acute{a})} g\big(a, f(a)\big)\right) = \bigoplus_{a \in A} g\big(a, f(a)\big)$$

Therefore we have the desired conclusion.

■

## Composition of morphisms

*Proposition: Composition of reactant morphisms*

Let $m \in (S,R) \to (\hat{S},\hat{R})$ and $\hat{m} \in (\hat{S},\hat{R}) \to (\bar{\bar{S}},\bar{\bar{R}})$ be reactant morphisms. Then $(\hat{m} \circ m) \in (S,R) \to (\bar{\bar{S}},\bar{\bar{R}})$ is a reactant morphisms.

*Proof*

Take any $\rho \to^k \pi \in R$. Since $m$ and $\hat{m}$ are reactant morphism we have that $\exists \hat{\pi},\hat{k}\;\; m(\rho \to^k \pi) = m(\rho) \to^{\hat{k}} \hat{\pi}$ and $\exists \bar{\bar{\pi}},\bar{\bar{k}}\;\; \hat{m}\big(m(\rho) \to^{\hat{k}} \hat{\pi}\big) = \hat{m}\big(m(\rho)\big) \to^{\bar{\bar{k}}} \bar{\bar{\pi}}$. Hence $\forall \rho \to^k \pi \in R\;\; \exists \bar{\bar{\pi}},\bar{\bar{k}}\;\; (\hat{m} \circ m)(\rho \to^k \pi) = (\hat{m} \circ m)(\rho) \to^{\bar{\bar{k}}} \bar{\bar{\pi}}$, and so $(\hat{m} \circ m) \in (S,R) \to (\bar{\bar{S}},\bar{\bar{R}})$ is a reactant morphism.

■

*Definition - Stoichiomorphisms*

$m \in (S,R) \to (\hat{S},\hat{R})$ is a CRN stoichiomorphisms iff

$$\forall s \in S\;\; \forall \hat{r} \in \hat{R}\;\; \textstyle\sum_{r \in m^{-1}(\hat{r})} \varphi(s,r) = \varphi(m(s),\hat{r})$$

*Proposition: Composition of stoichiomorphisms*

Let $m \in (S,R) \to (\hat{S},\hat{R})$ and $\hat{m} \in (\hat{S},\hat{R}) \to (\bar{\bar{S}},\bar{\bar{R}})$ be stoichiomorphisms. Then $(\hat{m} \circ m) \in (S,R) \to (\bar{\bar{S}},\bar{\bar{R}})$ is a stoichiomorphism.

*Proof*

We have:

$$\forall s \in S\;\; \forall \hat{r} \in \hat{R}\;\; \textstyle\sum_{r \in m^{-1}(\hat{r})} \varphi(s,r) = \varphi(m(s),\hat{r}) \qquad (Assumption\ 1)$$

$$\forall \hat{s} \in \hat{S}\;\; \forall \bar{\bar{r}} \in \bar{\bar{R}}\;\; \textstyle\sum_{\hat{r} \in \hat{m}^{-1}(\bar{\bar{r}})} \varphi(\hat{s},\hat{r}) = \varphi(\hat{m}(\hat{s}),\bar{\bar{r}}) \qquad (Assumption\ 2)$$

We need to show that the stoichiomorphism condition holds for $\hat{m} \circ m$:

$$\forall s \in S\;\; \forall \bar{\bar{r}} \in \bar{\bar{R}}\;\; \textstyle\sum_{r \in (\hat{m} \circ m)^{-1}(\bar{\bar{r}})} \varphi(s,r) = \varphi((\hat{m} \circ m)(s),\bar{\bar{r}})$$

Take any $s \in S$, $\bar{\bar{r}} \in \bar{\bar{R}}$; we can derive the above equality as follows:

$$\begin{aligned}
&\textstyle\sum_{r \in (\hat{m} \circ m)^{-1}(\bar{\bar{r}})} \varphi(s,r) \\
&= \textstyle\sum_{r \in m^{-1}\left(\hat{m}^{-1}(\bar{\bar{r}})\right)} \varphi(s,r) \\
&= \textstyle\sum_{\hat{r} \in \hat{m}^{-1}(\bar{\bar{r}})} \sum_{r \in m^{-1}(\hat{r})} \varphi(s,r) && \text{(see below)} \\
&= \textstyle\sum_{\hat{r} \in \hat{m}^{-1}(\bar{\bar{r}})} \varphi(m(s),\hat{r}) && \text{Assumption 1} \\
&= \varphi\big(\hat{m}(m(s)),\bar{\bar{r}}\big) && \text{Assumption 2} \\
&= \varphi\big((\hat{m} \circ m)(s),\bar{\bar{r}}\big)
\end{aligned}$$

By the Fiber Lemma, $\forall P,\hat{P}\;\; \forall m \in P \to \hat{P}\;\; \forall g \in P \times \hat{P} \to \mathbb{R}^+$ we have:

$$\textstyle\sum_{\hat{r} \in \hat{P}} \sum_{r \in m^{-1}(\hat{r})} g(r,\hat{r}) = \sum_{r \in P} g(r,m(r))$$

Given $s \in S$, $\bar{\bar{r}} \in \bar{\bar{R}}$, take $\hat{P} = \hat{m}^{-1}(\bar{\bar{r}})$, $P = m^{-1}(\hat{P})$, and we have $m \in P \to \hat{P}$. Take $g(x,y) = \varphi(s,x)$, then :

$$\textstyle\sum_{\hat{r} \in \hat{m}^{-1}(\bar{\bar{r}})} \sum_{r \in m^{-1}(\hat{r})} \varphi(s,r) = \sum_{r \in m^{-1}\left(\hat{m}^{-1}(\bar{\bar{r}})\right)} \varphi(s,r)$$

Which justifies the step above.

■

## Only-if propositions

*Lemma - Mass action (if direction) – previously proven.*

Let $m \in (S,R) \to (\hat{S},\hat{R})$ be a CRN morphism.

If $m$ is a CRN reactant morphism then $\forall \hat{\boldsymbol{v}} \in \mathbb{R}_+^{\hat{S}}\ \ \forall r \in R\ \ [r]_{\hat{\boldsymbol{v}}\circ m} = [m(r)]_{\hat{\boldsymbol{v}}}$.

*Lemma - Mass action (only-if direction)*

Let $m \in (S,R) \to (\hat{S},\hat{R})$ be a CRN morphism.

If $\forall \hat{\boldsymbol{v}} \in \mathbb{R}_+^{\hat{S}}\ \ \forall r \in R\ \ [r]_{\hat{\boldsymbol{v}}\circ m} = [m(r)]_{\hat{\boldsymbol{v}}}$, then $m$ is a CRN reactant morphism.

*Proof*

Assume $m \in (S,R) \to (\hat{S},\hat{R})$ is *not* a CRN reactant morphism; that means:

$$\exists r = \rho \to^{k} \pi \in R\ \ m(r) = \hat{\rho} \to^{\hat{k}} \hat{\pi}\ \ \ and\ \ \ \hat{\rho} \neq m(\rho)$$

The latter inequality means:

$$\exists \grave{s} \in \hat{S}\ \ \hat{\rho}_{\grave{s}} \neq m(\rho)_{\grave{s}}$$

Take $\hat{\boldsymbol{v}} \in \mathbb{R}^{+\hat{S}}$ such that $\hat{\boldsymbol{v}}_{\grave{s}} = 2$ and $\hat{\boldsymbol{v}}_{\hat{s}} = 1$ for all $\hat{s} \neq \grave{s}$. Then, using the Mass Action Lemma:

$$\begin{aligned}
&[m(r)]_{\hat{\boldsymbol{v}}} = \left[\hat{\rho} \to^{\hat{k}} \hat{\pi}\right]_{\hat{\boldsymbol{v}}} = \hat{\boldsymbol{v}}^{\hat{\rho}} = \textstyle\prod_{\hat{s}\in\hat{S}} \hat{\boldsymbol{v}}_{\hat{s}}{}^{\hat{\rho}_{\hat{s}}} = 2^{\hat{\rho}_{\grave{s}}} \\
&\neq 2^{m(\rho)_{\grave{s}}} = \textstyle\prod_{\hat{s}\in\hat{S}} \hat{\boldsymbol{v}}_{\hat{s}}{}^{m(\rho)_{\hat{s}}} = \hat{\boldsymbol{v}}^{m(\rho)} \\
&= (\hat{\boldsymbol{v}} \circ m)^{\rho} = [\rho \to^{k} \pi]_{\hat{\boldsymbol{v}}\circ m} = [r]_{\hat{\boldsymbol{v}}\circ m}
\end{aligned}$$

We have shown the contrapositive, that $\exists \hat{\boldsymbol{v}} \in \mathbb{R}_+^{\hat{S}}\ \ \exists r \in R\ \ [r]_{\hat{\boldsymbol{v}}\circ m} \neq [m(r)]_{\hat{\boldsymbol{v}}}$.

∎

*Remark – Alternate emulation theorems*

If $m$ preserves $\rho$ and $k$ (e.g., if it is a homomorphism) then $k \cdot (\hat{\boldsymbol{v}} \circ m)^{\rho} = m(k) \cdot \hat{\boldsymbol{v}}^{m(\rho)}$ where $k \cdot \boldsymbol{v}^{\rho}$ is what is normally understood as the instantanous mass action of a reaction. That consequence replaces the Mass Action Lemma and can be combined with the net stoichiometry condition ($\eta$ instead of $\varphi$) to obtain again an emulation theorem. But that is a more restrictive theorem from a stronger assumption that does nota allow $k$ to vary.

What if $m$ preserves only $k \cdot \rho$ (where $(k \cdot \rho)(s) = k \cdot \rho_s$) ? Then the following CRN satisfies that condition but is not an emulation: $m(s) = \hat{s}$ and $m(s \to^{2k}) = 2\hat{s} \to^{k}$, because $2k[s] \neq k[s]^2$. Note that this is a stoichiomorphism but is not a reactant morphism.

∎

*Theorem – Emulation (if direction) – previously proven*

Let $m \in (S,R) \to (\hat{S},\hat{R})$ be a CRN reactant morphism.
If $m$ is a CRN stoichiomorphism, then it is a CNR emulation.

*Theorem – Emulation (only-if direction)*

Let $m \in (S,R) \to (\hat{S},\hat{R})$ be a CRN reactant morphism and let $\hat{R}$ have no two reactions with the same reagents.
If $m$ is a CNR emulation then it is a CRN stoichiomorphism.

*Proof*

Take any $s \in S$. Since $m$ is an emulation we have $\forall \hat{\boldsymbol{v}} \in \mathbb{R}_+^{\hat{S}}\ F(\hat{\boldsymbol{v}} \circ m)(s) = \hat{F}(\hat{\boldsymbol{v}})(m(s))$, that is:

$$\forall \hat{\boldsymbol{v}} \in \mathbb{R}_+^{\hat{S}}\ \textstyle\sum_{r \in R}\ \varphi(s,r) \cdot [r]_{\hat{\boldsymbol{v}} \circ m} = \sum_{\hat{r} \in \hat{R}}\ \varphi(m(s),\hat{r}) \cdot [\hat{r}]_{\hat{\boldsymbol{v}}}$$

As in the proof of the Emulation Theorem (If direction) we obtain from the Fiber Lemma that:

$$\forall \hat{\boldsymbol{v}} \in \mathbb{R}_+^{\hat{S}}\ \textstyle\sum_{\hat{r} \in \hat{R}} \sum_{r \in m^{-1}(\hat{r})}\ \varphi(s,r) \cdot [\hat{r}]_{\hat{\boldsymbol{v}}} = \sum_{r \in R}\ \varphi(s,r) \cdot [m(r)]_{\hat{\boldsymbol{v}}}$$

And since $m$ is a reactant morphism, by the Mass Action Lemma, $\forall r \in R\ \ [m(r)]_{\hat{\boldsymbol{v}}} = [r]_{\hat{\boldsymbol{v}} \circ m}$, hence:

$$\forall \hat{\boldsymbol{v}} \in \mathbb{R}_+^{\hat{S}}\ \textstyle\sum_{\hat{r} \in \hat{R}} \sum_{r \in m^{-1}(\hat{r})}\ \varphi(s,r) \cdot [\hat{r}]_{\hat{\boldsymbol{v}}} = \sum_{r \in R}\ \varphi(s,r) \cdot [r]_{\hat{\boldsymbol{v}} \circ m}$$

Hence, from the emulation property above, and expanding $\hat{r} = \hat{\rho} \to^{\hat{k}} \hat{\pi}$:

$$\forall \hat{\boldsymbol{v}} \in \mathbb{R}_+^{\hat{S}}\ \textstyle\sum_{\hat{r} = \hat{\rho} \to^{\hat{k}} \hat{\pi} \in \hat{R}} \sum_{r \in m^{-1}(\hat{r})}\ \varphi(s,r) \cdot \hat{\boldsymbol{v}}^{\hat{\rho}} = \sum_{\hat{r} = \hat{\rho} \to^{\hat{k}} \hat{\pi} \in \hat{R}}\ \varphi(m(s),\hat{r}) \cdot \hat{\boldsymbol{v}}^{\hat{\rho}}$$

Take any $\hat{r} = \hat{\rho} \to^{\hat{k}} \hat{\pi} \in \hat{R}$: we can choose a $\hat{\boldsymbol{v}} \in \mathbb{R}_+^{\hat{S}}$ such that $\hat{\boldsymbol{v}}_{\hat{s}} = 0$ if $\hat{\rho}_{\hat{s}} = 0$ and $\hat{\boldsymbol{v}}_{\hat{s}} = 1$ otherwise, so that $\hat{\boldsymbol{v}}^{\hat{\rho}} = 1$ and $\hat{\boldsymbol{v}}^{\hat{\rho}'} = 0$ for any $\hat{\rho}' \neq \hat{\rho}$. Then for that $\hat{\boldsymbol{v}}$ only the terms with $\hat{\rho}$ as reagent are left as non-zero summands, each of those with $\hat{\boldsymbol{v}}^{\hat{\rho}} = 1$, reducing the sums to:

$$\textstyle\sum_{\hat{r} \in \hat{R}\ s.t.\ 1^{st}(\hat{r}) = \hat{\rho}} \sum_{r \in m^{-1}(\hat{r})}\ \varphi(s,r) = \sum_{\hat{r} \in \hat{R}\ s.t.\ 1^{st}(\hat{r}) = \hat{\rho}}\ \varphi(m(s),\hat{r})$$

By assumption, if each $\{\hat{r} \in \hat{R}\ s.t.\ 1^{st}(\hat{r}) = \hat{\rho}\}$ is a singleton, we obtain:

$$\textstyle\sum_{r \in m^{-1}(\hat{r})}\ \varphi(s,r) = \varphi(m(s),\hat{r})$$

Since this holds for any $s \in S$ and any $\hat{r} \in \hat{R}$, it means that $m$ is a stoichiomorphism.

■

*Remark – Generalization of emulation (only-if) theorem*

Under a generalization of CRNs to rational stoichiometric coefficients of reaction products, it is possible to normalize CRNs (while preserving kinetics) so that no two reactions have the same reagents. The Emulation (Only-If) Theorem then becomes a full if-and-only-if for such normalized CRNs.

■
